# Establishing a Working Definition of User Experience for eHealth Interventions of Self-reported User Experience Measures With eHealth Researchers and Adolescents: Scoping Review

**DOI:** 10.2196/25012

**Published:** 2021-12-02

**Authors:** Amanda S Newton, Sonja March, Nicole D Gehring, Arlen K Rowe, Ashley D Radomski

**Affiliations:** 1 Department of Pediatrics University of Alberta Edmonton, AB Canada; 2 School of Psychology and Counselling Centre for Health Research University of Southern Queensland Springfield Central Australia; 3 Knowledge Institute for Child and Youth Mental Health and Addictions Ottawa, ON Canada; 4 CHEO (Children’s Hospital of Eastern Ontario) Research Institute Ottawa, ON Canada

**Keywords:** eHealth, internet, design, development, user experience, health care, scoping review, Delphi

## Abstract

**Background:**

Across eHealth intervention studies involving children, adolescents, and their parents, researchers have measured user experience to assist with intervention development, refinement, and evaluation. To date, no widely accepted definitions or measures of *user experience* exist to support a standardized approach for evaluation and comparison within or across interventions.

**Objective:**

We conduct a scoping review with subsequent Delphi consultation to identify how user experience is defined and measured in eHealth research studies, characterize the measurement tools used, and establish working definitions for domains of user experience that could be used in future eHealth evaluations.

**Methods:**

We systematically searched electronic databases for published and gray literature available from January 1, 2005, to April 11, 2019. We included studies assessing an eHealth intervention that targeted any health condition and was designed for use by children, adolescents, and their parents. eHealth interventions needed to be web-, computer-, or mobile-based, mediated by the internet with some degree of interactivity. We required studies to report the measurement of *user experience* as first-person experiences, involving cognitive and behavioral factors reported by intervention users. We appraised the quality of user experience measures in included studies using published criteria: *well-established*, *approaching well-established*, *promising*, or *not yet established*. We conducted a descriptive analysis of how user experience was defined and measured in each study. Review findings subsequently informed the survey questions used in the Delphi consultations with eHealth researchers and adolescent users for how user experience should be defined and measured.

**Results:**

Of the 8634 articles screened for eligibility, 129 articles and 1 erratum were included in the review. A total of 30 eHealth researchers and 27 adolescents participated in the Delphi consultations. On the basis of the literature and consultations, we proposed working definitions for 6 main user experience domains: acceptability, satisfaction, credibility, usability, user-reported adherence, and perceived impact. Although most studies incorporated a study-specific measure, we identified 10 well-established measures to quantify 5 of the 6 domains of user experience (all except for self-reported adherence). Our adolescent and researcher participants ranked perceived impact as one of the most important domains of user experience and usability as one of the least important domains. Rankings between adolescents and researchers diverged for other domains.

**Conclusions:**

Findings highlight the various ways in which user experience has been defined and measured across studies and what aspects are most valued by researchers and adolescent users. We propose incorporating the working definitions and available measures of user experience to support consistent evaluation and reporting of outcomes across studies. Future studies can refine the definitions and measurement of user experience, explore how user experience relates to other eHealth outcomes, and inform the design and use of human-centered eHealth interventions.

## Introduction

### Background

Over the past 15 years, the number of eHealth interventions available for use by children, adolescents, and their parents has grown considerably. A commonly used approach to eHealth intervention development involves human-centered design (also known as patient- or user-centered design) [1,2]. This approach includes the active participation of intervention users—children, adolescents, and parents—in the intervention design and development process. By including user perspectives and input into intervention design, the likelihood that an intervention will be easy to use and be compatible with the user and their individual context, and therefore deemed useful, is improved [3-5]. More recently, the importance of users’ involvement in intervention evaluation has been recognized, with measures of *user experience* included in evaluations to identify whether and how an eHealth intervention meets the preferences and needs of the users. Understanding user experience can more or less reflect the quality of human-centered design principles associated with an intervention.

The term *user experience* initially arose in the field of human-computer interaction and technology design and was broadly defined as, “a person’s perception and responses that result from the use or anticipated use of a product, system or service” [6]. To date, across eHealth studies, a wide range of definitions and concepts have been used to evaluate user experience, such as satisfaction, acceptability, adherence, engagement, and usability, with an eHealth intervention [7-14]. Similarly, user experience data collection methods have also varied, such as with the use of self-report questionnaires, in-person or telephone-based interview guides, or different types of automatic data capture of users’ interactions with an intervention [15]. These variations suggest that user experience may be a multidimensional concept with several important constructs to define and measure within an eHealth intervention, and a consensus among researchers is yet to be reached. Similar to how the need to define, standardize, and measure adherence has been mounting in recent years [16,17], a need to converge on a common understanding of user experience is also becoming more apparent. A set of accepted domains, definitions, and evaluation measures used in eHealth intervention development and evaluation would benefit children, adolescents, and parents by allowing them to compare user experiences between multiple interventions and inform decisions about their own eHealth intervention use. These accepted approaches would also provide guidance to researchers in the eHealth field and allow for continued advancement and improvement of the study of user experience and other eHealth outcomes (eg, intervention effectiveness, user safety, and user empowerment), intervention design components (eg, content and technological features), and factors that can influence the intervention experience of users (eg, context of use and user expectations).

### This Study

This study involves two phases: a scoping review and Delphi consultations. Our decision to conduct a review plus consultation reflects a hermeneutic position that user experience cannot be fully understood without examining it in its current context (existing literature) and the meanings attributed to it (Delphi consultations). The scoping review includes diverse literature to identify how user experience has been defined and measured in eHealth research studies of children, adolescents, and parents. These findings subsequently informed the development of surveys used in the Delphi consultations with eHealth researchers and adolescent users of an eHealth intervention, which focused on establishing a working definition of user experience (and the domains that it may encompass) and developing recommendations for measuring user experience in future evaluations of eHealth interventions.

## Methods

### Study Design

We followed the scoping review framework proposed by Arksey and O’Malley [18] with a Delphi consultation recommended by Levac et al [19]. Reporting of the review adheres to the PRISMA (Preferred Reporting Items for Systematic Reviews and Meta-Analyses) Extension for Scoping Reviews checklist [20]. The consultation exercise followed synthesis of findings from the literature. Approvals from the Health Research Ethics Board at the University of Alberta and Human Research Ethics Committee at the University of Southern Queensland were received for the Delphi consultation. The approved study protocol is available upon request.

### Development of the Search Strategy

The search strategy (Multimedia Appendix 1) was developed using an iterative process. First, we developed a list of search terms using key concepts and terms from a convenience sample of indexed studies published in various years that examined user experience with an eHealth intervention. A research librarian provided input on appropriate filters, such as Medical Subject Headings terms, and modified these terms to comply with different databases. For this review, we were interested in identifying both published and unpublished English language studies of user experiences, and we sought to include studies made available between January 1, 2005, and April 11, 2019. We included the literature published since 2005 to focus on contemporary studies of eHealth technologies (eg, mobile apps, desktop-based, and multisession or single-session interventions). The search terms and parameters were tested for sensitivity, determined by whether the search strategy successfully filtered the 63 citations that were manually selected a priori (see Multimedia Appendix 2 for the list of test citations). We then conducted 2 rounds of preliminary screening to further refine the strategy. The finalized search strategy was peer-reviewed before implementation.

### Search Strategy

We identified studies from the following databases: Ovid MEDLINE, PsycINFO, CINAHL, EBM Reviews (Cochrane Database of Systematic Reviews, ACP Journal Club, Database of Abstracts of Reviews of Effects, Cochrane Central Register of Controlled Trials, Cochrane Methodology Register, Health Technology Assessment, and NHS Economic Evaluation Database), Cochrane Central Register of controlled trials, and ClinicalTrials.gov. We searched Google Scholar from January 2005 to April 2019 and conference proceedings of the International Society for Research on Internet Interventions from January 2016 to April 2019, as there are no archives of International Society for Research on Internet Interventions previous to 2016. We reviewed the reference lists from reviews (systematic, narrative, etc) to identify additional, potentially relevant studies.

### Criteria for Considering Studies for the Scoping Review

We included studies of any design that assessed user experience with an eHealth treatment or prevention intervention designed for children or adolescents (aged ≤19 years). Studies with a sample that contained young adults could be included in the review if the mean age of participants was reported to be ≤19 years. eHealth interventions could target any health condition but needed to be web-, computer-, or mobile-based, mediated by the internet and include some degree of interactivity. Studies of telehealth interventions were not included. Studies could assess the eHealth user experience of children, adolescents, or parents. Given the wide range of pre-existing definitions and measurement approaches used to evaluate eHealth intervention user experience, multiple domains could be included in the user experience evaluation, such as cognitive factors (ie, beliefs, attitudes, and intention; such as satisfaction and acceptability of the intervention) or behavioral factors (ie, how the intervention was used, such as self-reported adherence to and engagement with the intervention) related to the use of an eHealth intervention. To be included, studies needed to report the measurement of *user experience* as first-person experiences reported by eHealth intervention users (parents, children, and adolescents). Studies that only reported indirect user data (ie, proxy report by a parent whose child used a program and intervention metadata [number of sessions completed]), which do not reflect the user’s subjective experience, were excluded. Studies also needed to detail the evaluation measures (eg, tool, instrument, or interview questions) used to collect user experience data so that we could identify how user experience was defined and measured. Studies that did not detail evaluation questions but referenced an original publication of the evaluation measure were included if we could obtain the referenced publication to extract information.

### Screening for Article Eligibility

We organized and screened identified studies using EndNote (Clarivate Analytics) bibliographic management software. In pairs, 3 reviewers (authors NDG and AKR and review contributor Marcus O’Neill) independently screened the title and abstract of articles, classifying each as *relevant*, *irrelevant*, or *unclear* using the predetermined inclusion and exclusion criteria. To assess the clarity of the criteria for each reviewer during screening, we calculated the interrater agreement for screening outcomes for the first 100 articles using the κ statistic [21]. The agreement was *substantial* (κ=0.61). We wanted interrater agreement to be ≥0.80, indicating an *almost perfect* agreement [22], so reviewers met to review the screening criteria alongside the articles for which they disagreed and sought consensus on the screening outcome for each article. Agreement increased to *almost perfect* (κ=0.81) for the next set of 100 articles and therefore the screening progressed. At this point, reviewers divided the remaining articles to be screened. Articles screened as relevant or unclear underwent independent screening and discussion by each reviewer pair to determine study inclusion or exclusion. The reviewers contacted the primary authors of 9 articles when additional information was needed to determine eligibility. The reviewers documented the articles that were excluded after full-text review to ensure transparency and replicability.

### Data Extraction

#### Process

Data were extracted into a standardized spreadsheet. The spreadsheet underwent pilot testing with 3 independent reviewers (authors NDG and AKR and review contributor MO) who extracted data from the first 5 included studies to ensure that the spreadsheet was adequate in scope and that consensus was achieved on data categorization. Subsequently, each reviewer extracted data from one-third of the remaining included studies. Each reviewer verified the accuracy and completeness of the other reviewers’ respective thirds. Data extraction discrepancies were resolved through consensus and third-party consultation (author ASN). Corresponding authors were contacted when reporting was unclear or details were lacking in the article.

#### Data Extracted for Analysis

We extracted the following information from the studies:

1. General information on participants (age [range] and intervention [user or respondent]) and the eHealth intervention (name, mode of delivery, target population or health condition and duration or frequency).

2. How first-person user experience was defined in studies, which included looking for definitions and terms of user-reported experiences as well as extracting individual questions used to measure user experience. We then compared author-reported user experience definitions to a priori definitions and the identified measures were categorized into 6 domains: satisfaction, acceptability, credibility, impact, adherence, and use. The domains were based on a preliminary literature review of user experiences in eHealth studies that was conducted by one of the authors [23]. All tools fit into one or more of the 6 domains. The original working definitions for the 6 domains are presented in Multimedia Appendix 3.

3. Major characteristics of the evaluation measures used to assess user experience, including its purpose and scope, delivery time points, type of respondent (child, adolescent, or parent), administration approach (web-, telephone-, or paper-based or face-to-face interview), the number of items and item-response format (eg, Likert scale or open-ended questions), and any notations by study authors regarding limitations of the evaluation measures and recommendations for future measurement or evaluation.

4. Information on the measure’s psychometrics was extracted, if available, including information on measure validity (face, content, construct, or criterion), reliability (internal consistency, interrater, or test-retest), and findings from a factor analysis. In studies where an evaluation measure was referenced, the original reference was reviewed and psychometric data were extracted, if available.

### Quality Assessment

Two independent reviewers (NDG and AKR) assessed the quality of the evaluation measures reported in the studies and met to resolve discrepancies through consensus. The reviewers used 3 criteria developed by the Society for Pediatric Psychology Assessment Task Force [24]. The first criterion was the availability of details on the instrument or measure to allow evaluation and replication. This involved the reviewers confirming whether a measure was available for review in published or gray literature; study authors could have also included their measure as supplementary material to their publication. The second criterion concerned the availability of reliability and validity data for the instrument or measure. This could include psychometric data (eg, for surveys or rating scales) and data from pilot testing (eg, face and content validity or interview guide reliability for author-developed interviews). The third criterion, use of the instrument or measure by multiple, independent investigative teams as described in peer-reviewed articles, necessitated the measure or tool (including a qualitative interview guide) to have been used by more than one group.

Using the abovementioned criteria, measures were classified into 4 categories: *well-established*, *approaching well-established*, *promising*, or *not yet established*. We rated a measure as *well-established* if we could identify 2 peer-reviewed articles with very good detail of the measures and good to strong or excellent published information on both validity and reliability. We rated a measure as *approaching well-established* if we could identify 2 peer-reviewed articles with very good detail of the measure and with published information on validity *or* reliability either missing or presented in vague or poor to moderate values. We rated a measure as *promising* if we could identify 1 peer-reviewed article with sufficient detail of the measure (eg, some, but not all of the questions present) and with published information on validity *or* reliability either missing or presented in vague or poor to moderate values. Although not included in the original task force rating scheme, we rated a measure as *not yet established* if we could identify 1 peer-reviewed article with sufficient detail of the measure but with published validity or reliability information not available. The quality of validity and reliability data were interpreted using a guide presented by Phillips et al [25] (Multimedia Appendix 4).

### Data Analysis

Evidence tables and a bar graph were developed to aggregate findings into descriptive and thematic summaries [5]. Descriptive summaries include information on study, participant, and eHealth intervention characteristics, user experience measures, and related psychometric statistics. Thematic summaries include grouping measures according to the quality assessment categories used to define the measures: *well-established*, *approaching well-established*, *promising*, or *not yet established*.

### Delphi Consultation Process

The Delphi consultation phase of the scoping review was a stepwise process involving multiple, structured rounds of surveys to gain consensus [26-28] on how user experience should be defined and measured.

#### Participants

We sought input from 2 groups of individuals: researchers of the studies included in our review who had published eHealth intervention user experience evaluations and adolescents (aged 16-18 years) currently using an eHealth intervention.

We identified and contacted researcher participants using the published email contact information of lead or corresponding authors of studies included in the scoping review. We used snowball sampling so that contacted authors could also recommend colleagues with relevant expertise who may be interested in participating [26]. Each potential participant received an email invitation to participate along with an information sheet describing the Delphi consultation; those who completed the survey were considered to have given implied consent. The Delphi consultation with researchers was held over 7 months between September 2019 and March 2020.

For practical and feasibility reasons, we recruited adolescent participants among current users of an evidence-based eHealth intervention, the web-based BRAVE Self-Help program [12,29], who had previously consented to be contacted for future research studies. Recruitment involved a pop-up invitation that appeared when users logged into the BRAVE Self-Help program throughout a 6-month period from June 7, 2019, to December 10, 2019. Interested participants were directed to an external site and invited to read a separate information sheet regarding the study and provide informed consent to participate in the Delphi consultation. After providing consent on the web, participants completed a survey that was used for the Delphi consultation.

#### Process

**Figure 1:**
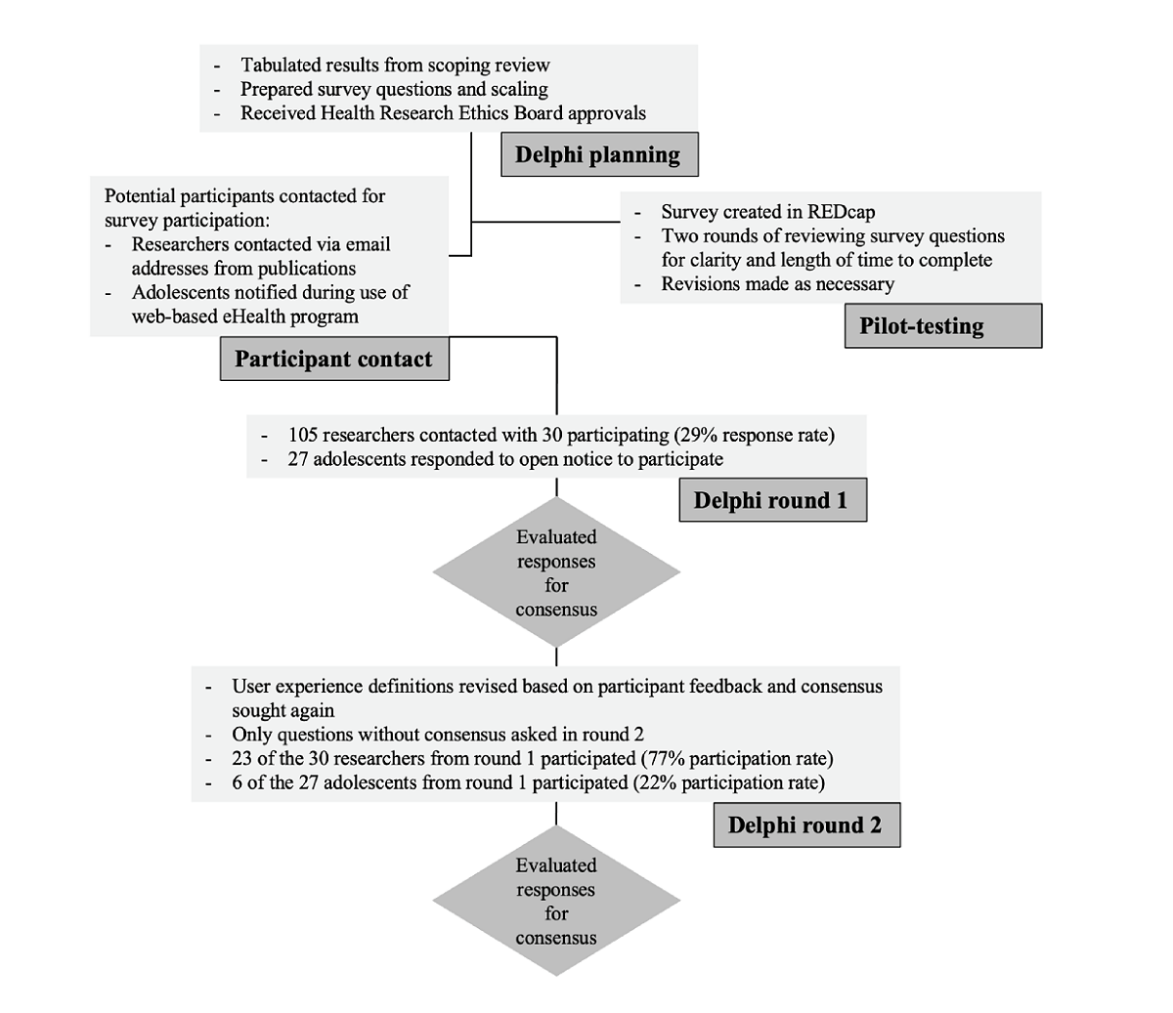
Delphi consultation process. REDCap: Research Electronic Data Capture.

The process that we followed with each participant group along with the response and participation rates is presented in Figure 1. Broadly, each survey included questions that sought consensus on participants’ opinions on the importance of the user experience domains and definitions used in the scoping review (Multimedia Appendix 3), additional domains for consideration, and the appropriateness of measures used across eHealth studies of user experience. Consensus on responses to each survey question was defined as having ≥80% agreement [26].

#### Survey Development and Scaling

Our team pilot-tested the surveys used in round 1 for both participant groups. We reviewed each survey for face validity, clarity, cohesiveness, flow, and completion time and made changes as needed (eg, modifying the wording of questions and changing the order of questions).

Researcher participants were first asked questions regarding their demography (age, gender, professional position, country of residence, and experience in developing and measuring user experience) followed by a series of questions on their level of agreement with the preliminary definitions of the proposed user experience domains (satisfaction, acceptability, credibility, impact, adherence, and use); initial definitions were based on a preliminary literature review that informed this scoping review as having accepted definitions or domains for measuring user experience. In round 1, an open text box for respondents to provide text supporting their answers was included. We originally wanted to survey participants for their opinions regarding the appropriateness of the user experience measures used across eHealth as well as findings from the review itself. We used a 6-point Likert scale ranging from *strongly agree* to *strongly disagree* to measure participants’ level of agreement. In round 1, we also included an open text box for suggestions and comments regarding proposed definitions of identified domains and for those domains not identified in the survey. For proposed domains that could be measured at multiple time points, we also asked participants to indicate their preferred timing of measurement (before, during, or after the intervention). Researcher participants were then asked to rank the importance of the domains relative to one another using a 6-point Likert scale ranging from *most important* to *least important* when measuring user experience. Participants were also asked in both rounds to indicate the importance of studies to measure user experience; however, we realized that definitions would need to be determined before this could occur.

Adolescent participants were asked questions regarding their demography (age, gender, and frequency of using web-based health interventions) followed by questions on their level of agreement with how important it is for researchers to ask them about each of the 6 user experience domains when using an eHealth intervention. Given the complexity and technical nature of the domains presented, adolescents were provided with lay descriptions of each of the domains and asked to rate how important they felt each domain was rather than commenting on the definition (as we did with the expert researcher sample). In this way, adolescents were able to provide input into the domains that were most important from their perspective. We used a 6-point Likert scale ranging from *extremely important* to *not at all important* to measure participants’ level of agreement and also included an open text box in round 1 so that participants could identify reasons for why they felt a particular domain was important or not. Adolescent participants were then asked to rank the importance of the domains relative to one another from *most important* to *least important*. In round 1, an open text box was available for adolescents to add any other comments they had on the presented domains and any other aspects of user experience they thought were important or missing.

#### Consultation Rounds

We conducted independent Delphi consultations with each participant group—2 rounds with researcher participants and 2 rounds with adolescent participants—using a survey tailored to each group. The same participants participated in both rounds as the intent was to gain consensus (agreement) within the 2 participant groups. Given the nature of the questions being asked, we felt that 2 rounds were sufficient to capture participants’ opinions. In round 2, we presented the summary of responses for survey questions where consensus was not achieved in round 1 or where comments and feedback for a survey question necessitated further clarification. This approach allowed each participant in round 2 to express their opinion after observing and reflecting on the opinions of other researchers or adolescents. The purpose of this approach was to decrease the degree of dispersion or increase the degree of consensus in participants’ answers. The consultation exercise concluded after 2 rounds irrespective of whether consensus was reached on all survey items.

We used REDCap (Research Electronic Data Capture), a secure web-based platform [30], to administer the electronic surveys to researcher participants and the University of Southern Queensland Survey Tool based on Lime Survey and hosted on secure University of Southern Queensland servers for adolescent participants. Participants spent 15-30 minutes (researchers) or 10-15 minutes (adolescents) to complete the survey in each round. To maximize the response rate for each round, nonrespondents were sent an email reminder about the survey after every 7 days until 3 contact attempts had been made. Participants answered questions anonymously so that individual opinions did not influence other participants’ opinions [26]. Adolescent participants who completed the surveys were given the opportunity to enter a draw to win one of the 10 vouchers valued at Aus $40 (US $28.92) to be drawn at completion of the study. Researchers who completed the surveys were given the opportunity to enter a draw to win a CAD $150 (US $118.14) electronic gift card.

#### Data Analysis

In round 1, we calculated the response rate for researcher participants only, as we were not able to determine the number of adolescent invitees from the open invitation to participate. In round 2, we calculated the participation rate for both researchers and adolescents using the denominator from round 1. For both rounds, we generated descriptive statistics (frequencies and percentages) to determine the level of agreement (consensus) among participants for each survey question. This involved grouping the responses at each end of the Likert scales (eg, grouping *strongly agree* with *agree* and *disagree* with *strongly disagree*). We also reviewed the responses to middle Likert categories (eg, *agree*/*disagree* and *slightly agree*/*slightly disagree*) to identify the range of opinions. Among researcher participants, following round 1, we collated the open-text answers or feedback on definitions and used this text to revise the definitions for the domains of user experience. We revised each domain definition using suggestions from participants irrespective of whether consensus was reached on the definition in round 1. The rationale was that the suggestions added critical details and improvements to each definition. The revised definitions were then presented for consensus in round 2 during which participants were asked to indicate their level of agreement with the revised definitions. We used IBM SPSS (version 26) for all analyses.

## Results

### Literature Search and Selection

**Figure 2:**
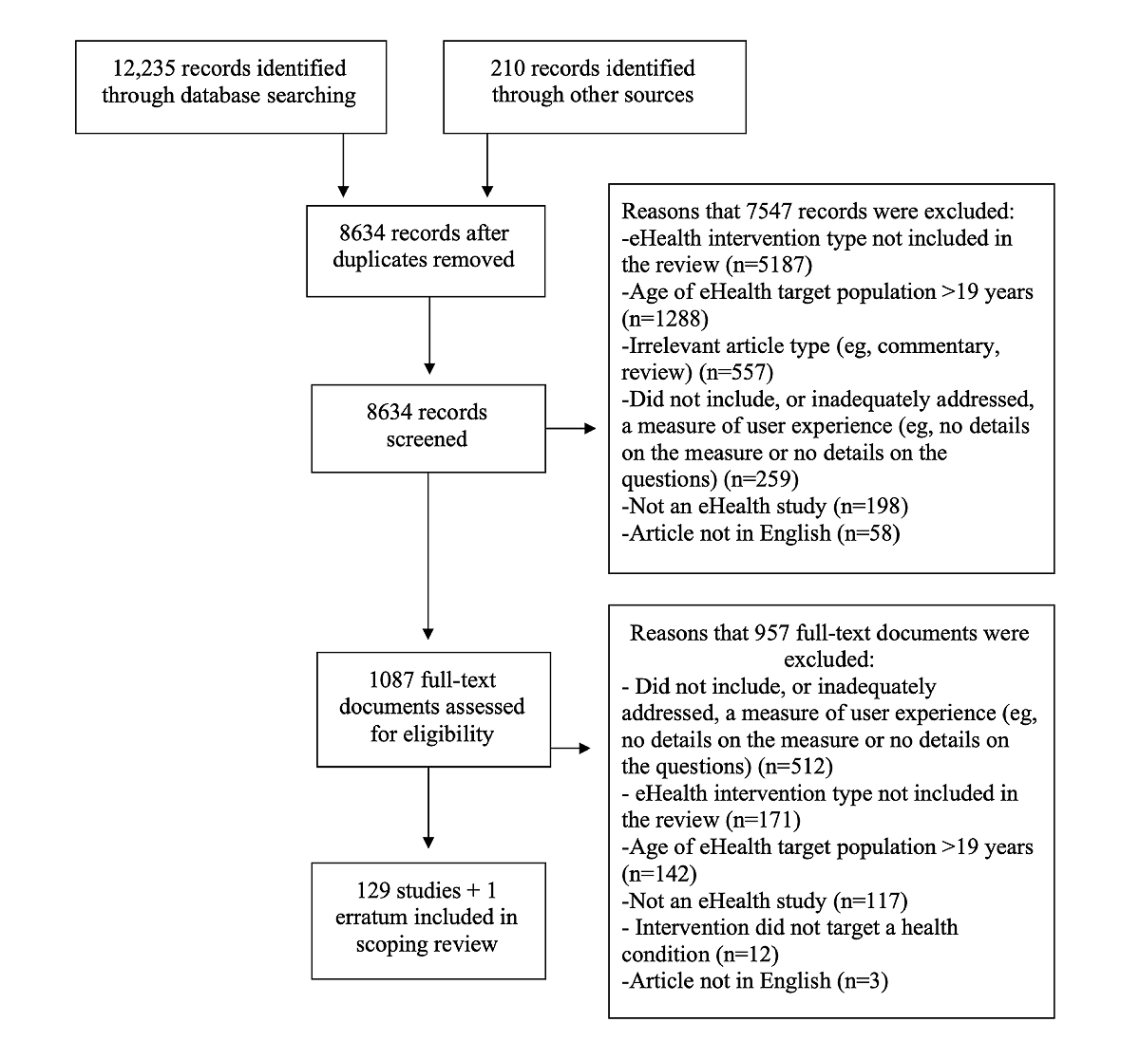
Literature search flow diagram.

The search strategy identified 8634 unique citations. Of these citations, 1087 were considered potentially relevant based on their title and abstract (Figure 2). After full-text review, 129 articles and 1 erratum met inclusion criteria and were included in the review.

### Description of Included Studies

**Figure 3:**
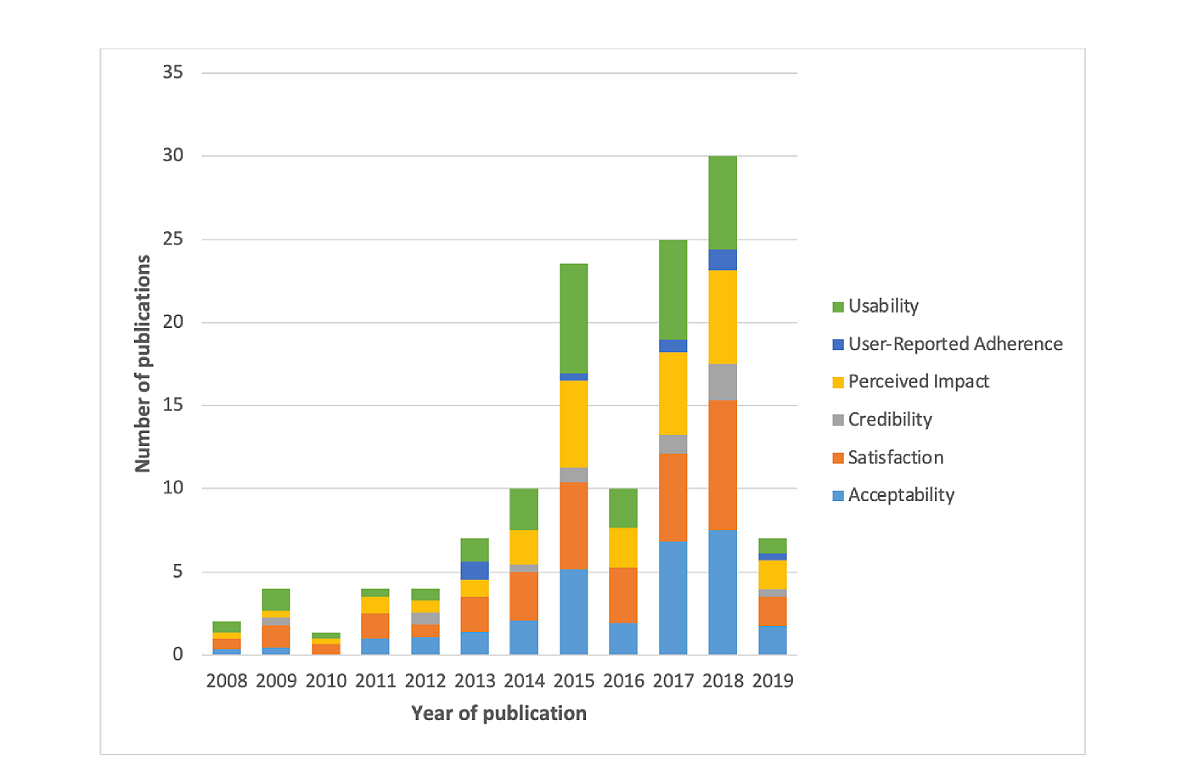
The type and frequency of user experience domains measured across eHealth studies over time. The domains named in this figure reflect the agreed-on terminology that resulted from the Delphi consultation with researchers.

A summary of the general characteristics of 129 eHealth studies that evaluated user experience is presented in Table 1. Most studies were published in 2018 (30/129, 23.3%), 2017 (25/129, 19.4%), and 2015 (24/129, 18.6%). The most commonly measured user experience domains were acceptability, usability, and satisfaction; this trend occurred across years of publication (Figure 3). Additional details for each study are presented in Multimedia Appendix 5 [7-14,31-152], grouped by year of publication to examine trends in the domains measured over time.

**Table 1:** Summary of the eHealth studies that measured user experience (N=129).

Characteristics			Studies within scoping review, n (%)
**eHealth user^a^**			
	Children aged ≤9 years	26 (20.2)	
	Adolescents aged 10-19 years	118 (91.5)	
	Young adults up to 24 years	25 (19.4)	
	Parents	33 (25.6)	
**Type of eHealth intervention**			
	Web-based	85 (65.9)	
	Mobile-based	44 (34.1)	
	Tablet-based	11 (8.5)	
**User experience domain that was measured**			
	Satisfaction	86 (66.7)	
	Acceptability	77 (59.7)	
	Credibility	17 (13.2)	
	Perceived impact	66 (51.2)	
	User-reported adherence	11 (8.5)	
	Usability	74 (57.4)	

^a^Age categories defined using World Health Organization definitions [153].

### User Experience Measures

Research teams used 128 unique user experience evaluation measures in the 129 published studies included in this review. Details of the quality assessment outcomes using the Society for Pediatric Psychology Assessment Task Force [24] criteria are provided in Multimedia Appendix 6 [7-14,31-111,113-121,123-152,154,155]. Of the 128 measures, 10 (7.8%) were assessed to be *well-established* measures (Table 2) and 5 (3.9%) were assessed to be *approaching well-established* (Table 3). These measures were used in research studies to primarily capture user experiences related to satisfaction with and acceptability of eHealth interventions and program usability and perceived impact of an intervention. Evaluation measures used in research studies that were assessed to be *promising* (13/129, 10.1%) are presented in Multimedia Appendix 7 [75,90,97,99,107,109,132,136,139,152]. The remaining 100 measures identified were assessed as *not yet established* and primarily represented studies in which author-developed evaluation questions were used. Information on evaluation measures used in research studies that were assessed to be *not yet established* is available upon request to the corresponding author.

**Table 2:** Well-established evaluation measures of user experience.

Measure name and targeted user experience domain	Format and administration features	Psychometric properties		eHealth study
		Validity	Reliability	
SUS^a^; usability	10 items; 4- and 5-point Likert scales; administration*:* paper- and web-based and telephonic	Two-factor scale [156]; usable (8 items), α=.91; learnable (2 items), α=.70; overall α=.92	Internal consistency; 10 years of SUS samples: α=.91 [157]; eHealth study sample: α=.86 [31], α=.95 [32]	[14,31-39]
SUS (Portuguese version); usability	10 items; 5-point Likert scale*;* administration: paper-based	Construct validity with PSSUQ^b^ [158]: *r*=0.70	Interrater reliability; Portuguese validation sample [158]: intraclass correlation coefficient=0.36 with modest agreement between ratings (76.67%)	[40]
Client Satisfaction Questionnaire 8; acceptability, satisfaction, and usability	8 items; 4-point Likert scale*;* administration: paper- and web-based	Criterion-related validity; other measures of satisfaction [159]: *r*=0.60-0.80	Internal consistency across 9 studies [159]: α=.83-.93; eHealth study sample: α=.92 [41]	[41-44]
CEQ^c^; credibility and perceived impact satisfaction	6 items; 9-point Likert scale and 0%-100% scale*;* administration: not reported	Two-factor scale [160]: expectancy (3 items), eigenvalue=3.42; credibility (3 items), eigenvalue=1.53; 2 factors accounted for 82.46% of the total variance	Internal consistency; CEQ validation across 3 studies [160]: expectancy, α=.79-.90; credibility, α=.81-.86; overall, α=.84-.85; test-retest reliability, CEQ validation across 3 studies [160]: expectancy, α=.82; credibility, α=.75	[41]
GEQ^d^; acceptability and satisfaction	33 items; 5-point Likert scale*;* administration: web-based	Five-factor scale; GEQ validation study [161]: factor 1 (5 items); factor 2 (7 items); factor 3 (4 items); factor 4 (5 items); factor 5 (4 items); all correlation coefficients >0.30	Internal consistency; GEQ development sample [162]: α=.81	[7,10]
PSSUQ also known as the Computer Systems Usability Questionnaire^e^; acceptability, perceived impact, satisfaction, and usability	19 items; 7-point Likert scale*;* administration: web-based	Three-factor scale; correlation coefficients on 5 years of PSSUQ samples [154]: system usefulness and informational quality, *r*=0.70; system usefulness and interface quality, *r*=0.70; informational quality and interface quality, *r*=0.60; factors scores shared 36% to 50% of the variance	Internal consistency; 5 years of PSSUQ samples [154]: system usefulness, α=.96; informational quality, α=.92; interface quality, α=.83; overall, α=.96	[45,46]
SSS^f^; satisfaction	5 items; 4-point Likert scale and open ended*;* administration: telephonic	One-factor scale factor loadings^g^ [163]: Youth version, α=.77-.90; Parent version, α=.71-.83	Internal consistency; SSS development samples [163]: youth α=.86; parent α=.85; eHealth study sample across different time points [47]: α=.77-.95	[47]
TEI-SF^h^; acceptability and perceived impact satisfaction	9 items; 5-point Likert scale*;* administration: paper-based	2 factor scale [164]: acceptability (8 items), α=.49-.93, 57% of total item variance; discomfort (1 item), α=.82, 12% of total item variance	Internal consistency; TEI-SF development samples: α=.94 [165], α=.85 [164]	[48]
USE^i^ questionnaire; acceptability, satisfaction, and usability	19 items (4 subscales); 7-point Likert scale*;* administration: web-based	Criterion-related validity [166]; compared with SUS (2 evaluations); usefulness: *r*_1_=0.60, *r*_2_=0.69; ease of learning: *r*_1_=0.71, *r*_2_=0.78; ease of use: *r*_1_=0.78, *r*_2_=0.81; satisfaction: *r*_1_=0.66, *r*_2_=0.71	Internal consistency; USE development sample [166]: α=.98; eHealth study sample [32]: usefulness, α=.90; ease of learning, α=.98; ease of use, α=.95; satisfaction, α=.96	[32]
WAI-SR^j^; credibility and perceived impact	12 items; 5-point Likert scale*;* administration: web-based	Three-factor scale; correlation with WAI-SR within 2 samples (S1 and S2) [167]: goal (4 items), α=.89 (S1), α=.87 (S2); task (4 items), α=.87 (S1), α=.90 (S2); bond (4 items), α=.86 (S1), α=.84 (S2); overall, α=.95 (S1), α=.94 (S2)	Internal consistency; WAI-SR development within 2 samples [167]: goal, α=.87 (S1), α=.85 (S2); task, α=.85 (S1), α=.87 (S2); bond, α=.90 (S1), α=.85 (S2); overall, α=.91 (S1), α=.92 (S2); eHealth study sample [41]: α=.95	[41,49]

^a^SUS: System Usability Scale.

^b^PSSUQ: Poststudy System Usability Questionnaire.

^c^CEQ: Credibility Expectancy Questionnaire.

^d^GEQ: Game Experience Questionnaire.

^e^The Computer Systems Usability Questionnaire and PSSUQ are the same questionnaire; the only difference is that the Computer Systems Usability Questionnaire wording is appropriate for use in field settings or surveys rather than in a scenario-based usability evaluation [154,155].

^f^SSS: Satisfaction with Services Scale.

^g^Factor loading: correlation coefficient for the variable and factor.

^h^TEI-SF: Treatment Evaluation Inventory-Short Form.

^i^USE: Usefulness, Satisfaction, and Ease of use.

^j^WAI-SR: Working Alliance Inventory: Revised Short form.

**Table 3:** Evaluation measures assessed to be approaching well-established.

Measure name and targeted user experience domain	Format and administration features	Psychometric properties		eHealth study
		Validity	Reliability	
Client Satisfaction Scale; perceived impact and satisfaction	10 items; 5-point Likert scale*;* administration*:* not reported	Not reported	Internal consistency; eHealth study sample [50]: Child scale, α=.75; Parent scale, α=.85	[50-52]
Standardized SUMI^a^; acceptability, satisfaction, and usability	55 items; 3-point Likert scale and open ended*;* administration*:* paper-based	Not reported	Internal consistency; SUMI development sample [168]: global subscale, α=.92; efficiency subscale, α=.81; affect subscale, α=.85; helpfulness subscale, α=.83; control subscale, α=.71; learnability subscale, α=.82	[53]
WAMMI^b^; acceptability and usability	20 items; 5-point Likert scale*;* administration: not reported	Not reported	Internal consistency; WAMMI development sample [169]: α=.96	[54]
Author-adapted TEI-SF^c^; acceptability and satisfaction	11 items; 5-point Likert scale*;* administration: paper-based	Not reported for author adaption of TEI-SF	Internal consistency; eHealth study sample [55]: Child scale, α=.82; Parent scale, α=.81	[55,56]
Author-developed questionnaire; acceptability and perceived impact	7 items; 5-point Likert scale*;* administration: web-based	One-factor scale [57]: 69% of total item variance	Internal consistency; eHealth study sample [57]: α=.94	[57]
Author-developed questionnaire; perceived impact and usability	>14 items; 5- and 10-point Likert scales*;* administration: not reported	Not reported	Internal consistency; eHealth study sample [58]: perceived benefits of intervention content, α=.92; perceived benefits of the interpersonal principles in the intervention, α=.85; ease of use, α=.94; ease of understanding, α=.96; ease of reading, α=.97; internal rationale, α=.96; identification/relevance, α=.96	[58,59]
Author-developed questionnaire; perceived impact, satisfaction, and usability	7 items; 4-point Likert scale*;* administration: not reported	Two-factor scale: utility of program (5 items), 45% of total item variance; user friendliness of program (2 items), 21% of total item variance	Internal consistency; eHealth study sample [60]: user friendliness, α=.71; utility items, α=.84	[60]
Author-developed questionnaire; acceptability, satisfaction, and perceived impact	17 items; scale type not reported*;* administration: not reported	Three-factor scale; eHealth study sample [61]: program evaluation (7 items); program benefits (7 items); overall satisfaction (3 items); subscales ranged from 1 (negative evaluation) to 10 (positive evaluation)	Internal consistency; eHealth study sample [61]: subscales, α=.90 or higher	[61]

^a^SUMI: Software Usability Measurement Inventory.

^b^WAMMI: Website Analysis and Measurement Inventory.

^c^TEI-SF: Treatment Evaluation Inventory- Short Form.

### Delphi Consultation

#### Overview

In round 1, 30 researchers and 27 adolescents participated. In round 2, the number of participants decreased to 23 researchers (23/30, 77% participation rate) and 6 adolescents (6/27, 22% participation rate; Figure 1). The demographic characteristics of researcher and adolescent participants are presented in Table 4. Researcher participants were mainly women and employed in academic positions, and all participants had used a measure of user experience in their work. In addition, most adolescent participants were female participants, and most had limited experience in using eHealth interventions (despite being currently enrolled in an eHealth intervention for anxiety).

**Table 4:** Demographic information about the participants.

Characteristics				Round 1		Round 2
**Researcher participants, n (%)**				30 (100)		23 (77)
	Age (years), mean (SD)			42.6 (1.4)		43.7 (1.7)
	**Sex, n (%)**					
		Female	26 (86.7)		20 (87)	
		Male	4 (13.3)		3 (13)	
	**Primary role or position, n (%)**					
		Academic (professor and lecturer)	19 (63.3)		13 (56.5)	
		Scientist (researcher and research fellow)	5 (16.7)		5 (21.7)	
		Clinician	4 (13.3)		4 (17.4)	
		Trainee (PhD candidate and postdoctoral fellow)	2 (6.7)		1 (4.3)	
	**Country, n (%)**					
		Australia	4 (12.9)		3 (13)	
		Canada	3 (9.7)		2 (8.7)	
		Finland	1 (3.2)		1 (4.3)	
		Ireland	2 (6.5)		1 (4.3)	
		Italy	1 (3.2)		1 (4.3)	
		Korea	1 (3.2)		1 (4.3)	
		New Zealand	4 (12.9)		3 (13)	
		Sweden	2 (6.5)		2 (8.7)	
		United Kingdom	1 (3.2)		1 (4.3)	
		United States	11 (35.5)		8 **(**34.8)	
	Has measured user experience, n (%)			30 (100)		23 (100)
	Has developed a user experience measure, n (%)			7 (23.3)		6 (26.1)
**Adolescent participants, n (%)**				27 (100)		6 (22)
	Age (years), mean (SD)			16.44 (0.6)		N/A^a,b^
	**Sex, n (%)**					
		Female	20 (74.1)		N/A	
		Male	5 (18.5)		N/A	
		Other	2 (7.4)		N/A	
	**Use of eHealth programs, n (%)**					
		Never used until the day of the survey	14 (51.9)		N/A	
		<once per week	7 (25.9)		N/A	
		1-2 times per week	4 (14.8)		N/A	
		3-4 times per week	0 (0)		N/A	
		5-6 times per week	1 (3.7)		N/A	
		≥7 times per week	1 (3.7)		N/A	

^a^N/A: not applicable.

^b^Demographics for adolescent participants in round 2 were not collected to ensure anonymity as per the research ethics board’s requirements.

#### Researcher Participants

Over 2 rounds of consultation, researcher participants made several suggestions for refining the original definition of each user experience domain. The revised definitions achieved by round 2 are presented in Table 5 along with the consensus scores for the definition (the percentage of participants who *strongly agreed* or *agreed* with the definition). Researchers met or surpassed the threshold for agreement on all definitions except the definition for perceived impact. Regarding the importance of each of the domains to the overall assessment of user experience in round 1, researchers achieved consensus in their ranking of credibility as less important to measure relative to the other 5 domains (80%; Table 6); no other rankings achieved consensus in this round. Perceived impact was ranked in both rounds as more important than the other domains, but consensus (≥80% agreement) was not achieved. Regarding when assessment of user experience should be conducted, the responses differed across domains. By round 2, researchers agreed that eHealth intervention acceptability (87% consensus) and satisfaction (97% consensus) should be measured after intervention completion. Researchers were divided on when credibility should be measured, with equal proportions of researchers indicating credibility should be measured at all 3 time points (before the intervention: 61%; during the intervention: 65%; and after the intervention: 61%).

**Table 5:** Working definitions of user experience domains developed with researcher participants.^a^

Domain	User experience definition	Definition consensus (%)
Acceptability	Acceptability refers to whether the intervention *content, features, and delivery* meet user *expectations* (eg, relevance, convenience, accessibility, feasibility, appropriateness, *appeal [fun, interesting, and likable], value, engaging, and privacy*). *These aspects may be different depending on the user (ie, child, adolescent, or parent).*	100
Satisfaction	Satisfaction refers to *the user’s overall impression of the* intervention *and whether it meets their needs* (eg, global satisfaction rating, *value for money or time, helpful, whether they would they use it again or recommend it to a friend, and ratio between expectations and results*).	96
Credibility	Credibility refers to the extent to which the user perceives the intervention to be trustworthy and *has the potential to work* (eg, perceived accuracy and quality of information *in the intervention and/or evidence base supporting the intervention*).	96
Usability^b^	Usability refers to the user’s perceived ease of use *of the intervention based on technical factors (eg, interface/equipment/reminder features or problems)* and environmental/personal factors (eg, content, time, and *competing priorities*) *that impact* the individual’s use of the intervention (eg, *frequency*).	87
User-reported adherence^c^	User-reported adherence refers to how and why the user *did or did not follow* the intervention or research protocol (eg, completing outcome measures and content) as recommended.^d^	83
Perceived impact	Perceived impact refers to the extent to which the user *perceives the effect of the intervention’s impacts (eg, impressions of* change in symptom levels and skills and *perception* of overall effectiveness).	78

^a^Italics represent additions or changes to the original definition. Domains are listed in descending order of consensus.

^b^Usability should also be measured in conjunction with objective measures of use (ie, intervention metadata).

^c^User-reported adherence should also be measured in conjunction with objective measures of adherence (ie, intervention metadata) and clinician expectations and adherence to the protocol (if relevant).

^d^Content removed from the definition.

**Table 6:** The relative rankings among researcher participants for the importance of the user experience domains across 2 rounds of consultation.

Domain	Round 1 (%)		Round 2 (%)	
	More important	Less important	More important	Less important
Acceptability	73	27	48	52
Satisfaction	43	57	30	70
Credibility	20	80	—^a^	—
Usability	47	53	26	74
User-reported adherence	47	53	26	74
Perceived impact	70	30	65	35

^a^Round 2 not conducted as consensus was achieved in round 1.

Researcher participants’ opinions varied greatly on whether a universal measure of user experience was needed to enable direct comparisons between studies of eHealth interventions. By round 2, a total of 70% (16/23) stated that a universal measure was extremely or quite important, 22% (5/23) stated that it was slightly important, and 9% (2/23) stated it was slightly unimportant. In round 1, a total of 37% (11/30) of the participants also provided comments. Most stated that although a universal measure may be impractical given variability across users’ developmental stage, technology types, and language, a core set of user experience items would be a valuable addition to the eHealth field. Participants suggested that other measures could be added to this core set to obtain intervention-specific feedback as needed. Moreover, 1 participant pointed out that accepted definitions of user experience domains are also important to ensure that the domains are not used differently from study to study even if different measures are used to assess them.

#### Adolescent Participants

Adolescents were asked to indicate which user experience domains were important for a researcher to ask them about. In round 1, adolescents reached consensus that acceptability was more important for a researcher to ask about compared with other domains (81% consensus); satisfaction and perceived impact were indicated as important, but adolescents did not reach consensus in round 1 (78% consensus for both). After the second round, adolescents achieved consensus with satisfaction, credibility, and perceived impact (100% consensus) and usability (83% consensus) identified as important domains for researchers to ask about when measuring adolescents’ user experience. After the 2 survey rounds, adolescents remained divided on the importance of measuring user-reported adherence, with 50% (3/6) of the participants rating it more important and 50% (3/6) rating it as less important

When asked to rank the domains in order of importance to one another (ie, which domains were more important than the other domains), perceived impact was considered the most important domain to measure (83% consensus) based on 2 rounds of consultation. Credibility and acceptability were also ranked highly and were considered more important than the other domains, but they did not meet or surpass the 80% threshold to achieve consensus. User-reported adherence was ranked the least important domain relative to the others (83% consensus). Usability (76%) and satisfaction (67%) were also ranked as less important to measure, although consensus was not reached for these 2 domains either.

## Discussion

### Summary of Principal Findings

To our knowledge, this is the first study to review how eHealth intervention studies have defined and measured user experience, consult with experts (researchers) as to how user experience could be defined and measured, and consult with users (adolescents) as to which domains they considered important for examining their user experience. In the scoping review, we made 2 important discoveries: several well-established measures are available to quantify user experiences and a large proportion of published eHealth studies did not involve the use of a well-established measure, with authors having developed user experience questions specific to their eHealth intervention. Key findings from our Delphi consultation are the alignment between researcher and adolescent relative rankings of user experience domains and the refinement of definitions for the 6 proposed domains of user experience.

### Discussion of Principal Findings

We identified 10 well-established measures available in the current literature to quantify 5 of the 6 proposed domains of user experience (satisfaction, acceptability, credibility, perceived impact, and usability). Therefore, we recommend that eHealth researchers use an available, well-established measure instead of developing their own measure to assess these 5 user experience domains. This approach will allow for between-study comparisons of user experiences, including whether and how experiences with similar eHealth interventions are considered the same or different among users. Not only could such comparisons help inform parents and their children with their decisions for which eHealth intervention to use but also findings across studies could be used by researchers to identify and understand the relationship between user experience and other eHealth outcomes (ie, intervention effectiveness and program adoption or use).

To date, no well-established measure exists for studying user-reported adherence. This is not surprising, as adherence is typically measured using intervention metadata, such as the number of sessions completed and time spent per session. We propose that in addition to such objective metrics, researchers measure how and why a user *did or did not follow* the intervention or research protocol (eg, completing outcome measures and content) as recommended or as encouraged by intervention design. This subjective information can expand the understanding of metadata—for example, why did the user complete the number of sessions or outcome measures that they did. These data could be collected using a set of open-ended questions designed to be used across studies and tailored, when needed, to specific interventions, studies, or user profiles.

Definitions of the 6 domains of user experience that resulted from our international consultation with eHealth researchers offer a guidepost for new studies of user experience. Although the proposed definitions may continue to be refined over time as the eHealth field advances, we see them as an important cornerstone to user experience measurement. A set of commonly accepted domains, similar to the efforts to define, standardize, and measure eHealth adherence [16,17] and engagement [170,171], can introduce a taxonomy that can be applied across eHealth studies even if different populations, interventions, and measures are used. However, it remains unclear, as to whether a universal measure of user experience would be useful for researchers. Although 16 of the 23 of the researchers in this study responded that it is important to use a universal measure, several concerns were brought forward regarding the challenge to implementing such an approach. As a follow-up to the results we reported here, further investigation of the utility and feasibility of a core set of items and what this core set should be is needed. It is possible that consideration needs to be given to how measures can be adapted for different respondents (eg, parents, adolescents, and clinicians) or different intervention contexts (eg, open access vs therapist supported). For example, the findings of this study showed that perceived impact was rated as important by both the researchers and adolescents; however, it is entirely possible that each would describe the desired impact of the intervention in different ways. Further investigation is necessary to examine how these constructs can be best assessed using different respondents and contexts.

The Delphi consultations we conducted provide insight into the value that researchers and adolescents place on the different domains of a user’s experience with an eHealth intervention. Both adolescent and researcher respondents ranked perceived impact as one of the most important aspects of a user’s eHealth experience, indicating that whether the user perceives the intervention to have had an impact on their health is a central component of user experience. There were also divergent perspectives, with one the most divergent being researchers’ ranking of satisfaction among the most important domains compared with adolescents’ ranking of it as less important. At the core of the definition of satisfaction is an emphasis on whether an intervention meets a user’s expectations and needs. From the adolescent point of view, the definition of perceived impact, which focuses on the *impressions of change in symptom levels and skills or the perception of overall effectiveness*, may have been a more meaningful way to identify whether their needs would be met, as perceived impact directly links to an observable or tangible change in their symptoms or behavior because of the eHealth program. The ranking of credibility also differed between researchers (lower ranking) and adolescents (higher ranking). With the choice of eHealth interventions increasing, as consumers, it is not surprising that adolescents would place greater priority on credibility as an important aspect to the selection and use of an eHealth intervention. This prioritization should be considered by researchers when developing and marketing their interventions for use.

Usability was considered one of the least important domains of user experience from the perspective of both adolescent and researcher respondents. This ranking may reflect the discrepancy between the more commonly used definitions of usability and the one proposed in our Delphi consultation. Originating from the field of computer science, usability is considered a main design component (design heuristic), similar to aesthetics, user safety, or data privacy, meaning that a certain degree of (technical or functional) usability is fundamental to or expected with the use of an eHealth intervention. Although an intervention may be usable, it does not mean that it will be *used* or perceived as *useful* by adolescents [172]. Therefore, if usability is currently understood as a measure of the technical ease of use or the functionality of an intervention, then examining it may add little *richness* to the understanding of what and how users describe their user experience with eHealth intervention to be like. However, if usability comes to be associated with the conditions (barriers or constraints, facilitators, and context) of use and reasons for usefulness (meets the needs and preferences of users), then usability may be considered an important indicator of the user experience.

### Future Directions

This scoping review with Delphi consultations has provided a broad overview of the current state of user experience measurement in the eHealth field along with expert (researcher) and user (adolescent) input into how user experience could be defined and measured with an eHealth intervention. An extension of this work may include investigating whether a core set of items used to measure the various domains of user experience would add value to the field and could be feasibly applied across different interventions and user populations [23]. Future research could benefit from qualitative investigations with adolescents to further define their understanding and definitions of the user experience domains within different eHealth contexts and test the feasibility of core assessment items for these domains from their perspective. It would then be beneficial to validate their definition of user experience (and the associated domains) and test the feasibility of core measurement item sets across large numbers of adolescent users of eHealth interventions internationally. With greater awareness and emphasis on patient-oriented research and improving outcomes important to patients, assessment of user experience can become an important part of patient-centered treatment planning.

Further attention could also be directed toward the definition and measurement of self-reported adherence. In our study, although a definition of self-reported adherence was achieved through consensus, its importance in measurement was not established among researcher or adolescent participants. Although a wealth of measurement studies exists for understanding adherence from an objective standpoint [16,173,174], few explanatory studies have been conducted to explore how and why the user did or did not follow the intervention and research protocol as recommended [23]. This *why* component is critical for meaningful improvement of eHealth interventions to increase program adherence and therefore achieve related health benefits [175].

Future studies may also look at how to apply both objective and subjective intervention outcomes or user experience measures to improve the validity of eHealth evaluations. For example, adolescents and researchers in our study reported perceived impact to be an important aspect of the user experience. Objective measures of intervention impact, such as changes in diagnostic severity, global functioning, symptom checklists [176], or self-reported minimal clinically important difference [177], could reinforce or complement the findings generated by more subjective measures [23]. In this way, we could better understand how various measures converge or diverge on similar user experience concepts, begin to develop more psychometrically and theoretically robust assessment measures, and establish indicators of clinically meaningful outcomes based on users’ perspectives.

### Limitations

Although this scoping review and associated Delphi consultations were conducted according to published guidelines, this study is not without limitations. First, our study focus was placed on eHealth interventions that were web-, computer-, or mobile-based and mediated by the internet; we excluded studies that did not primarily include these features and therefore, our results will not be representative of all technologies for which user experience may be measured. In addition, eHealth interventions being used and evaluated in health care systems that have not been scientifically investigated and reported in the published or gray literature were not included in our review. Our scoping review focus also required studies to describe the measure or measures used to collect user experience data. This requirement resulted in the exclusion of 512 studies. Given that this was a review of definition and measurement of user experience, such details were essential to understanding the current state of the eHealth field. This approach was systematic in that we applied the same working definitions to each study; however, it may have resulted in the classification of a domain of user experience that differed from what study investigators intended. The challenge in grouping some of the studies confirms that agreement regarding definitions of user experience domains would be of value to the eHealth field. The proposed domains and definitions are not intended to be static, and we expect that they will be refined to reflect advances in the eHealth field. Finally, although we present the results from the first international Delphi consultations, our sample size was limited; particularly in representation from adolescent users.

### Conclusions

eHealth interventions are now widely available for use by children, adolescents, and parents, and positive user experiences are generally reported across individual studies. The outcomes of this review and Delphi process highlight the various ways in which user experience has been defined and measured across studies, with a large proportion of research studies using study-specific, nonstandardized instruments. Through the conduct of this study, we propose definitions for 6 user experience domains: acceptability, satisfaction, credibility, usability, user-reported adherence, and perceived impact, as informed by empirical literature and agreed upon by eHealth researchers and adolescent users. Findings revealed 10 well-established measures that assess 5 of the 6 user experience domains (satisfaction, acceptability, credibility, perceived impact, and usability), and we recommend that eHealth researchers use an available, well-established measure over developing their own to assess these domains. The proposed working definitions and importance rankings from researcher and adolescent participants can be used to inform eHealth user experience research in the future and encourage consistency in reporting and to guide the development of measurement tools. Future studies should examine whether a core set of items used to measure the various domains of user experience would add value to the field and be feasibly applied across different interventions and user populations. Closing these gaps has the potential to enable comparisons across the user experience literature and better understand how user experience relates to other outcomes, such as effectiveness or objective measures of adherence, in eHealth interventions.

## Appendix

Multimedia Appendix 1 Search strategy for Ovid MEDLINE Epub ahead of print, in-process, and other nonindexed citations; Ovid MEDLINE Daily; and Ovid MEDLINE.

Multimedia Appendix 2 The list of 63 test citations that were used to develop search terms for the search strategy used to identify studies on user experiences.

Multimedia Appendix 3 The definitions of user experience domains used for data extraction in the scoping review.

Multimedia Appendix 4 The parameters used for reviewing and interpreting psychometric data during the quality assessment of evaluation measures (adapted from a published table [<xref ref-type="bibr" rid="ref25">25</xref>]).

Multimedia Appendix 5 eHealth studies that measured user experience published from 2008 to 2019.

Multimedia Appendix 6 Results from the quality assessment of user experience measures used in the 129 studies included in the review.

Multimedia Appendix 7 Evaluation measures used in the eHealth studies assessed to be promising.

## Abbreviations

PRISMA: Preferred Reporting Items for Systematic Reviews and Meta-Analyses
REDCap: Research Electronic Data Capture
