# Supplementary material for: Establishing a Working Definition of User Experience for eHealth Interventions of Self-reported User Experience Measures With eHealth Researchers and Adolescents: Scoping Review
Source: J Med Internet Res. 2021 Dec 2;23(12):e25012. doi: 10.2196/25012 (PMC8686463; doi:10.2196/25012)
Supplement: Multimedia Appendix 1 [file jmir_v23i12e25012_app1.docx]

## Multimedia Appendix 1

Search strategy for Ovid MEDLINE(R) Epub Ahead of Print, In-Process & Other Non-Indexed Citations, Ovid MEDLINE(R) Daily and Ovid MEDLINE(R)

| 1 exp Cell Phone/ut [utlization] (1076)  2 "Cell Phone Use"/ (17)  3 *Computer-Assisted Instruction/ (8403)  4 Computers/ut [utlization] (836)  5 *Internet/ and (diar* or intervention* or portal* or program* or site*).tw,kf. (10706)  6 Mobile Applications/ (2680)  7 Online Systems/ (7921)  8 Therapy, Computer-Assisted/ (6204)  9 ((app or application* or apps) adj2 (iPhone* or online* or mobile* or smartphone* or web*)).tw,kf. (7770)  10 ((computer* or electronic* or internet* or online* or web*) adj3 (diar* or portal* or program* or site*)).tw,kf. (33907)  11 ((computer* or electronic* or internet* or online* or web*) adj3 (intervention* or therap* or treatment* or prevent*)).tw,kf. (10714)  12 (e-health* or ehealth*).tw,kf. (4296)  13 (e-medic* or emedic*).tw,kf. (215)  14 (e-mental health* or emental health*).tw,kf. (163)  15 (e-p?ediatric* or ep?ediatric*).tw,kf. (9)  16 (e-psychiatr* or epsychiatr*).tw,kf. (9)  17 (e-therap* or etherap*).tw,kf. (679)  18 electronic communic*.tw,kf. (1201)  19 (information adj2 web*).ti. (251)  20 (m-health* or mhealth*).tw,kf. (2521)  21 (m-mental health* or mmental health*).tw,kf. (5)  22 mobile health*.tw,kf. (2395)  23 mobile phone based*.tw,kf. (389)  24 patient portal*.tw,kf. (573)  25 wearable*.tw,kf. (6352)  26 or/1-25 [Combined MeSh & text words for eHealth] (90380)  27 Adolescent/ (1844381)  28 exp Adolescent Behavior/ (26609)  29 Adolescent Health/ (502)  30 Adolescent Health Services/ (5149)  31 Child/ (1549548)  32 Child Behavior/ (15987)  33 Psychology, Adolescent/ (12833)  34 adolescen*.tw,kf. (251720)  35 child*.tw,kf. (1264901)  36 (school age* or schoolage* or schoolchild*).tw,kf. (30999)  37 teen*.tw,kf. (27138)  38 or/27-37 [Combined MeSH & text words for children & adolescents] (3070177)  39 Feasibility Studies/ (57056)  40 Feedback/ (28202)  41 Focus Groups/ (23797)  42 Follow-Up Studies/ (585134)  43 Interviews as Topic/ (53042)  44 Patient Compliance/ (53404)  45 exp Patient Satisfaction/ (77581)  46 Pilot Projects/ (104591)  47 Program Evaluation/ (55761)  48 Software Design/ (5626)  49 Self Report/ (21588)  50 User-Computer Interface/ (34136)  51 ((accept* or accessib* or experien* or friendl* or prefer* or satisf* or suitab*) adj5 (adolescen* or child* or participa* or patient* or user*)).tw,kf. (362269)  52 ((assess* or evaluat* or feedback* or rate* or rating* or report* or test*) adj2 (patient* or self* or user*)).tw,kf. (523652)  53 ((assess* or evaluat* or feedback* or rate* or rating* or report* or test*) adj3 (effective* or efficac*)).tw,kf. (234998)  54 ((assess* or evaluat* or feedback* or measure* or rate* or rating* or report* or test*) adj3 engage*).tw,kf. (3741)  55 ((assess* or evaluat* or feedback* or rate* or rating* or test*) adj3 (interface* or pilot* or program* or prototype*)).tw,kf. (61149)  56 ((challeng* or difficult* or eas* or intuiti* or simpl*) adj2 (navigat* or "use")).tw,kf. (34002)  57 (co-creat* or cocreat*).tw,kf. (529)  58 (co-design* or codesign*).tw,kf. (413)  59 credib*.tw,kf. (12706)  60 feasibil*.ti. (25557)  61 feasibil*.ab. /freq=2 (13890)  62 usab*.tw,kf. (17370)  63 usage*.tw,kf. (78858)  64 or/39-63 [Combined MeSH & text words for user experience] (2046634)  65 and/26,38,64 [Combined concepts for ehealth, adolescents & user experience] (5139)  66 exp Animals/ not Humans/ (4430733)  67 65 not 66 [Exclude animal studies] (5139)  68 (comment or editorial or news or newspaper article).pt. (1213278)  69 67 not 68 [Exclude opinion pieces] (5121)  70 ("2008 *" or "2009 *" or 201*).dt. (10406524)  71 69 and 70 [Limit date from 2008-Present] (3942)  72 remove duplicates from 71 (3933) |
| --- |
