# Supplementary material for: Establishing a Working Definition of User Experience for eHealth Interventions of Self-reported User Experience Measures With eHealth Researchers and Adolescents: Scoping Review
Source: J Med Internet Res. 2021 Dec 2;23(12):e25012. doi: 10.2196/25012 (PMC8686463; doi:10.2196/25012)
Supplement: Multimedia Appendix 2 [file jmir_v23i12e25012_app2.docx]

## Multimedia Appendix 2

The list of 63 test citations that were used to develop search terms for the search strategy used to identify studies of user experiences.

1. Abbott JA, Kaldo V, Klein B, Austin D, Hamilton C, Piterman L, et al. A cluster randomised trial of an internet-based intervention program for tinnitus distress in an industrial setting. Cogn Behav Ther 2009;38(3):162-73. PMID: 19675959

2. Abeles P, Verduyn C, Robinson A, Smith P, Yule W, Proudfoot J. Computerized CBT for adolescent depression (“Stressbusters”) and its initial evaluation through an extended case series. Behav Cogn Psychother 2009 Mar;37(2):151-65. PMID: 19364416

3. Aflague TF, Boushey CJ, Guerrero RT, Ahmad Z, Kerr DA, Delp EJ. Feasibility and use of the mobile food record for capturing eating occasions among children ages 3-10 years in Guam. Nutrients 2015;7(6):4403-15. PMID: 26043037

4. Alblas EE, Folkvord F, Anschütz DJ, Ketelaar PE, Granic I, Mensink F, et al. User statistics for an online health game targeted at children. Games Health J 2017;6(5):319-325. PMID: 28767272

5. Ammerlaan JJ, Scholtus LW, Drossaert CH, van Os-Medendorp H, Prakken B, Kruize AA, et al. Feasibility of a website and a hospital-based online portal for young adults with juvenile idiopathic arthritis: Views and experiences of patients. JMIR Res Protoc 2015;4(3):e102. PMID: 26276373

6. Ammerlaan J, van Os-Medendorp H, Scholtus L, de Vos A, Zwier M, Bijlsma H, et al. Feasibility of an online and a face-to-face version of a self-management program for young adults with a rheumatic disease: experiences of young adults and peer leaders. Pediatr Rheumatol Online J 2014;12:10. PMID: 24666817

7. Ammerlaan JW, van Os-Medendorp H, de Boer-Nijhof N, Maat B, Scholtus L, Kruize AA, Bijlsma JW, Geenen R. Preferences and needs of patients with a rheumatic disease regarding the structure and content of online self-management support. Patient Educ Couns 2017;100(3):501-508. PMID: 27776789

8. An LC, Schillo BA, Saul JE, Wendling AH, Klatt CM, Berg CJ, et al. Utilization of smoking cessation informational, interactive, and online community resources as predictors of abstinence: Cohort study. J Med Internet Res 2008;10(5):e55. PMID: 19103587

9. Andersson E, Enander J, Andrén P, Hedman E, Ljótsson B, Hursti T, et al. Internet-based cognitive behaviour therapy for obsessive-compulsive disorder: A randomized controlled trial. Psychol Med 2012;42(10):2193-203. PMID: 22348650

10. Andersson E, Hedman E, Wadström O, Boberg J, Andersson EY, Axelsson E, et al. Internet-based extinction therapy for worry: A randomized controlled trial. Behav Ther 2017;48(3):391-402. PMID: 28390501

11. Andrews JA, Gordon JS, Hampson SH, Gunn B, Christiansen SM, Slovic P. Long-term efficacy of click city(r): tobacco: A school-based tobacco prevention program. Nicotine Tob Res 2014;16(1):33-41. PMID: 23884322

12. Armbrust W, Bos JJ, Cappon J, van Rossum MA, Sauer PJ, Wulffraat N, et al. Design and acceptance of Rheumates@Work, a combined internet-based and in person instruction model, an interactive, educational, and cognitive behavioral program for children with juvenile idiopathic arthritis. Pediatr Rheumatol Online J 2015;13:31. PMID: 26202161

13. Arnaud N, Baldus C, Elgán TH, De Paepe N, Tønnesen H, Csémy L, et al. Effectiveness of a web-based screening and fully automated brief motivational intervention for adolescent substance use: A randomized controlled trial. J Med Internet Res. 2016 May 24;18(5):e103. PMID: 27220276

14. Arnaud N, Bröning S, Drechsel M, Thomasius R, Baldus C. Web-based screening and brief intervention for poly-drug use among teenagers: Study protocol of a multicentre two-arm randomized controlled trial. BMC Public Health 2012;12:826. PMID: 23013141

15. Arnold RJ, Stingone JA, Claudio L. Computer-assisted school-based asthma management: A pilot study. JMIR Res Protoc 2012;1(2):e15. PMID: 23612058

16. Breakey VR, Ignas DM, Warias AV, White M, Blanchette VS, Stinson JN. A pilot randomized control trial to evaluate the feasibility of an Internet-based self-management and transitional care program for youth with haemophilia. Haemophilia 2014;20(6):784-93. PMID: 25311370

17. Breakey VR, Warias AV, Ignas DM, White M, Blanchette VS, Stinson JN. The value of usability testing for Internet-based adolescent self-management interventions: “Managing Hemophilia Online”. BMC Med Inform Decis Mak 2013;13:113. PMID: 24094082

18. Britto MT, Jimison HB, Munafo JK, Wissman J, Rogers ML, Hersh W. Usability testing finds problems for novice users of pediatric portals. J Am Med Inform Assoc 2009;16(5):660-9. PMID: 19567793

19. Cafazzo JA, Casselman M, Hamming N, Katzman DK, Palmert MR. Design of an mHealth app for the self-management of adolescent type 1 diabetes: A pilot study. J Med Internet Res 2012;14(3):e70. PMID: 22564332

20. Cai RA, Beste D, Chaplin H, Varakliotis S, Suffield L, Josephs F, et al. Developing and evaluating JIApp: Acceptability and usability of a smartphone app system to improve self-management in young people with juvenile idiopathic arthritis. JMIR Mhealth Uhealth 2017;5(8):e121. PMID: 28811270

21. Comer JS, Furr JM, Cooper-Vince CE, Kerns CE, Chan PT, Edson AL, et al. Internet-delivered, family-based treatment for early-onset OCD: A preliminary case series. J Clin Child Adolesc Psychol 2014;43(1):74-87. PMID: 24295036

22. Cordova D, Alers-Rojas F, Lua FM, Bauermeister J, Nurenberg R, Ovadje L, et al. The usability and acceptability of an adolescent mHealth HIV/STI and drug abuse preventive intervention in primary care. Behav Med 2016:1-12. PMID: 27223646

23. Crosby LE, Ware RE, Goldstein A, Walton A, Joffe NE, Vogel C, et al. Development and evaluation of iManage: A self-management app co-designed by adolescents with sickle cell disease. Pediatr Blood Cancer 2017;64(1):139-145. PMID: 27574031

24. Dexheimer JW, Kurowski BG, Anders SH, McClanahan N, Wade SL, Babcock L. Usability evaluation of the SMART application for youth with mTBI. Int J Med Inform. 2017;97:163-170. PMID: 27919376

25. Hetrick SE, Dellosa MK, Simmons MB, Phillips L. Development and pilot testing of an online monitoring tool of depression symptoms and side effects for young people being treated for depression. Early Interv Psychiatry 2015;9(1):66-9. PMID: 24612591

26. Hicks CL, von Baeyer CL, McGrath PJ. Online psychological treatment for pediatric recurrent pain: a randomized evaluation. J Pediatr Psychol. 2006 Aug;31(7):724-36. PMID: 16093516.

27. Hoek W, Schuurmans J, Koot HM, Cuijpers P. Effects of Internet-based guided self-help problem-solving therapy for adolescents with depression and anxiety: a randomized controlled trial. PLoS One 2012;7(8):e43485. PMID: 22952691

28. Holtz BE, Murray KM, Hershey DD, Richman J, Dunneback JK, Vyas A, Wood MA. The design and development of MyT1DHero: A mobile app for adolescents with type 1 diabetes and their parents. J Telemed Telecare 2019; 25(3):172-180. PMID: 29228854

29. Hundert AS, Huguet A, Green CR, Hewitt AJ, Mushquash CJ, Muhajarine N, et al. Usability testing of guided Internet-based parent training for challenging behavior in children with Fetal Alcohol Spectrum Disorder (Strongest Families FASD). J Popul Ther Clin Pharmacol 2016;23(1):e60-76. PMID: 27115205

30. Iloabachie C, Wells C, Goodwin B, Baldwin M, Vanderplough-Booth K, Gladstone T, et al. Adolescent and parent experiences with a primary care/Internet-based depression prevention intervention (CATCH-IT). Gen Hosp Psychiatry 2011;33(6):543-55. PMID: 21958447

31. Jibb LA, Cafazzo JA, Nathan PC, Seto E, Stevens BJ, Nguyen C, et al. Development of a mHealth real-time pain self-management app for adolescents with cancer: An iterative usability testing study. J Pediatr Oncol Nurs 2017;34(4):283-294. PMID: 28376666

32. Khanna MS, Kendall PC. Computer-assisted cognitive behavioral therapy for child anxiety: Results of a randomized clinical trial. J Consult Clin Psychol 2010;78(5):737-45. PMID: 20873909

33. Korus M, Cruchley E, Stinson JN, Gold A, Anthony SJ. Usability testing of the Internet program: “Teens Taking Charge: Managing My Transplant Online”. Pediatr Transplant 2015;19(1):107-17. PMID: 25495484

34. Kurowski BG, Wade SL, Dexheimer JW, Dyas J, Zhang N, Babcock L. Feasibility and potential benefits of a web-based intervention delivered acutely after mild traumatic brain injury in adolescents: A pilot study. J Head Trauma Rehabil 2016;31(6):369-378. PMID: 26360000

35. Lenhard F, Andersson E, Mataix-Cols D, Rück C, Vigerland S, Högström J, et al. Therapist-guided, Internet-delivered cognitive-behavioral therapy for adolescents with obsessive-compulsive disorder: A randomized controlled trial. J Am Acad Child Adolesc Psychiatry 2017;56(1):10-19.e2. PMID: 27993223

36. Lillevoll KR, Vangberg HC, Griffiths KM, Waterloo K, Eisemann MR. Uptake and adherence of a self-directed internet-based mental health intervention with tailored e-mail reminders in senior high schools in Norway. BMC Psychiatry 2014;14:14. PMID: 24443820

37. Lin HC, Chiang LC, Wen TN, Yeh KW, Huang JL. Development of online diary and self-management system on e-Healthcare for asthmatic children in Taiwan. Comput Methods Programs Biomed 2014;116(3):299-310. PMID: 24947614

38. Mulvaney SA, Anders S, Smith AK, Pittel EJ, Johnson KB. A pilot test of a tailored mobile and web-based diabetes messaging system for adolescents. J Telemed Telecare 2012;18(2):115-8. PMID: 22383802

39. Mulvaney SA, Rothman RL, Wallston KA, Lybarger C, Dietrich MS. An internet-based program to improve self-management in adolescents with type 1 diabetes. Diabetes Care 2010;33(3):602-4. PMID: 20032275

40. Newcombe PA, Dunn TL, Casey LM, Sheffield JK, Petsky H, Anderson-James S, et al. Breathe Easier Online: Evaluation of a randomized controlled pilot trial of an Internet-based intervention to improve well-being in children and adolescents with a chronic respiratory condition. J Med Internet Res 2012;14(1):e23. PMID: 22356732

41. Nieto R, Hernández E, Boixadós M, Huguet A, Beneitez I, McGrath P. Testing the feasibility of DARWeb: An online intervention for children with functional abdominal pain and their parents. Clin J Pain 2015;31(6):493-503. PMID: 25551478

42. Nijhof SL, Bleijenberg G, Uiterwaal CS, Kimpen JL, van de Putte EM. Effectiveness of internet-based cognitive behavioural treatment for adolescents with chronic fatigue syndrome (FITNET): A randomised controlled trial. Lancet 2012;379(9824):1412-8. PMID: 22385683

43. Nordfeldt S, Hanberger L, Berterö C. Patient and parent views on a Web 2.0 Diabetes Portal--the management tool, the generator, and the gatekeeper: Qualitative study. J Med Internet Res 2010;12(2):e17. PMID: 20511179

44. O’Malley G, Dowdall G, Burls A, Perry IJ, Curran N. Exploring the usability of a mobile app for adolescent obesity management. JMIR Mhealth Uhealth 2014;2(2):e29. PMID: 25098237

45. Pretorius N, Arcelus J, Beecham J, Dawson H, Doherty F, Eisler I, et al. Cognitive-behavioural therapy for adolescents with bulimic symptomatology: The acceptability and effectiveness of internet-based delivery. Behav Res Ther 2009;47(9):729-36. PMID: 19515360

46. Pretorius N, Rowlands L, Ringwood S, Schmidt U. Young people’s perceptions of and reasons for accessing a web-based cognitive behavioural intervention for bulimia nervosa. Eur Eat Disord Rev 2010;18(3):197-206. PMID: 20443203

47. Rhee H, Allen J, Mammen J, Swift M. Mobile phone-based asthma self-management aid for adolescents (mASMAA): A feasibility study. Patient Prefer Adherence 2014;8:63-72 PMID: 24470755

48. Sage A, Roberts C, Geryk L, Sleath B, Tate D, Carpenter D. A self-regulation theory-based asthma management mobile app for adolescents: A usability assessment. JMIR Hum Factors 2017;4(1):e5. PMID: 28148471

49. Shellmer DA, Dew MA, Mazariegos G, DeVito Dabbs A. Development and field testing of Teen Pocket PATH(®), a mobile health application to improve medication adherence in adolescent solid organ recipients. Pediatr Transplant 2016;20(1):130-40. PMID: 26916967

50. Sousa P, Fonseca H, Gaspar P, Gaspar F. Usability of an internet-based platform (Next.Step) for adolescent weight management. J Pediatr 2015;91(1):68-74. PMID: 25245364

51. Starling R, Nodulman JA, Kong AS, Wheeler CM, Buller DB, Woodall WG. Usability testing of an HPV information website for parents and adolescents. Online J Commun Media Technol 2015;5(4):184-203. PMID: 26594313

52. Stinson J, Gupta A, Dupuis F, Dick B, Laverdière C, LeMay S, et al. Usability testing of an online self-management program for adolescents with cancer. J Pediatr Oncol Nurs 2015;32(2):70-82. PMID: 25037173

53. Stinson JN, Jibb LA, Nguyen C, Nathan PC, Maloney AM, Dupuis LL, et al. Development and testing of a multidimensional iPhone pain assessment application for adolescents with cancer. J Med Internet Res 2013;15(3):e51. PMID: 23475457

54. Stinson J, McGrath P, Hodnett E, Feldman B, Duffy C, Huber A, et al. Usability testing of an online self-management program for adolescents with juvenile idiopathic arthritis. J Med Internet Res 2010;12(3):e30. PMID: 20675293

55. Stinson JN, Petroz GC, Tait G, Feldman BM, Streiner D, McGrath PJ, et al. e-Ouch: Usability testing of an electronic chronic pain diary for adolescents with arthritis. Clin J Pain 2006;22(3):295-305. PMID: 16514331

56. Trautmann E, Kröner-Herwig B. A randomized controlled trial of Internet-based self-help training for recurrent headache in childhood and adolescence. Behav Res Ther 2010;48(1):28-37. PMID: 19782343

57. Van Voorhees BW, Fogel J, Pomper BE, Marko M, Reid N, Watson N, et al. Adolescent dose and ratings of an Internet-based depression prevention program: A randomized trial of primary care physician brief advice versus a motivational interview. J Cogn Behav Psychother 2009;9(1):1-19. PMID: 20694059

58. Voerman JS, Remerie S, Westendorp T, Timman R, Busschbach JJ, Passchier J, et al. Effects of a guided Internet-delivered self-help intervention for adolescents with chronic pain. J Pain 2015;16(11):1115-26. PMID: 26281947

59. Voncken-Brewster V, Moser A, van der Weijden T, Nagykaldi Z, de Vries H, Tange H. Usability evaluation of an online, tailored self-management intervention for chronic obstructive pulmonary disease patients incorporating behavior change techniques. JMIR Res Protoc 2013 Jan 16;2(1):e3. PMID: 23612363

60. Whitehouse SR, Lam PY, Balka E, McLellan S, Deevska M, Penn D, et al. Co-creation with TickiT: Designing and evaluating a clinical eHealth platform for youth. JMIR Res Protoc 2013;2(2):e42. PMID: 24140595

61. Wilson M, Ramsay S, Young KJ. Engaging overweight adolescents in a health and fitness program using wearable activity trackers. J Pediatr Health Care 2017;31(4):e25-e34. PMID: 28501356

62. Wozney L, Baxter P, Newton AS. Usability evaluation with mental health professionals and young people to develop an Internet-based cognitive-behaviour therapy program for adolescents with anxiety disorders. BMC Pediatr 2015;15:213. PMID: 26675420

63. Ybarra ML, Bull SS, Prescott TL, Birungi R. Acceptability and feasibility of CyberSenga: An Internet-based HIV-prevention program for adolescents in Mbarara, Uganda. AIDS Care 2014;26(4):441-7. PMID: 24093828
