# Supplementary material for: Establishing a Working Definition of User Experience for eHealth Interventions of Self-reported User Experience Measures With eHealth Researchers and Adolescents: Scoping Review
Source: J Med Internet Res. 2021 Dec 2;23(12):e25012. doi: 10.2196/25012 (PMC8686463; doi:10.2196/25012)
Supplement: Multimedia Appendix 3 [file jmir_v23i12e25012_app3.docx]

## Multimedia Appendix 3

The definitions of user experience domains used for data extraction in the scoping review.

| **Domain** | **Definition** |
| --- | --- |
| Acceptability | From a user experience perspective, acceptability refers to whether the intervention met user needs (e.g., helpful, relevant, comprehensive, convenient, accessible, feasible, appropriate). |
| Satisfaction | From a user experience perspective, satisfaction refers to satisfaction with the intervention, such as with its content, features, and delivery (e.g., interactivity, aesthetics, ‘quality of care’), and/or the user’s overall impression of the intervention (i.e., global satisfaction rating). |
| Credibility | From a user experience perspective, credibility refers to the extent to which the user perceived the intervention to be logical, trustworthy, and based on information from authorities or reliable sources (e.g., confidence in treatment, perceived accuracy and quality of information). |
| Perceived Impact | From a user experience perspective, impact means the extent to which the user found the intervention beneficial to their health-related goal (e.g., contributed to skill development, improved self-management or feelings of empowerment, supported change in symptom levels, overall effectiveness). |
| User-reported Adherence | From a user experience perspective, adherence refers to how the user rated/described their adherence to the intervention and/or research protocol (e.g., filling out outcome measures). Adherence is rated/described relative to personal expectations (e.g., intrinsic expectations such as personal goals for treatment) and/or experiences (e.g., competing demands, symptoms). |
| Usability | From a user experience perspective, use refers to perceived ease of use (i.e., usability) and/or the impact of specific factors (e.g., intervention content, technical features, environmental influences, personal factors) on an individual’s use of the intervention. |
