# Supplementary material for: Establishing a Working Definition of User Experience for eHealth Interventions of Self-reported User Experience Measures With eHealth Researchers and Adolescents: Scoping Review
Source: J Med Internet Res. 2021 Dec 2;23(12):e25012. doi: 10.2196/25012 (PMC8686463; doi:10.2196/25012)
Supplement: Multimedia Appendix 4 [file jmir_v23i12e25012_app4.docx]

## Multimedia Appendix 4

The parameters used for reviewing and interpreting psychometric data during the quality assessment of evaluation measures (adapted from published table [25]).

| **Factor analysis** | | |
| --- | --- | --- |
| Suitability of data for factor analysis | | |
| Correlation coefficients | >0.30 |  |
| **Cronbach’s alpha** | | |
| Acceptability | Alpha value | Quality |
| Inadequate | 0.00–0.69 | Poor |
| Adequate | 0.70–0.79  0.80–0.89 | Moderate |
|  |  | Good |
| High | ≥0.90 | Excellent |
| **Pearson’s r, Spearman’s rho, Cohen’s kappa** | | |
| Correlation coefficient value | Quality | |
| ≤0.10 | No correlation |  |
| 0.11–0.29 | Poor |  |
| 0.30–0.49 | Low |  |
| 0.50–0.69 | Moderate |  |
| 0.70–0.89 | Strong |  |
| ≥0.90 | Very strong |  |
| **Intraclass correlation coefficient** | | |
| ICC value | Quality | |
| 0.45–0.75 | Fair to good |  |
| >0.75 | Excellent |  |
