# Supplementary material for: Establishing a Working Definition of User Experience for eHealth Interventions of Self-reported User Experience Measures With eHealth Researchers and Adolescents: Scoping Review
Source: J Med Internet Res. 2021 Dec 2;23(12):e25012. doi: 10.2196/25012 (PMC8686463; doi:10.2196/25012)
Supplement: Multimedia Appendix 5 [file jmir_v23i12e25012_app5.docx]

## Multimedia Appendix 5

eHealth studies that measured user experience published from 2008 to 2019.

| **First author** | **Intervention** | **Users** | **User experience domain that was measured** |
| --- | --- | --- | --- |
| **2019** | | | |
| Bauer [87] | ProYouth  *Features:* web-based  *Type of use:* self-led (one group clinician guided) | 12–18 year olds at risk of an eating disorder | Acceptability  Perceived Impact Satisfaction |
| Coulter [7] | No name; to improve help seeking and coping  *Features:* web-based  *Type of use:* self-led | 14–18 year olds who are of sexual and gender minority | Acceptability  Satisfaction |
| Guagliano [8] | FRESH to improve physical activity  *Features:* web-based; 1x/week over 6 weeks  *Type of use:* self-led + parental involvement | 8–10 year olds | Acceptability  Usability  User-Reported Adherence |
| Liu [41] | LYNX to increase HIV/STI testing and PrEP uptake  *Features:* mobile-based  *Type of use:* self-led | 15–24 year old men who have sex with men | Credibility  Perceived Impact |
| Nieto [88] | DARWeb  *Features:* web-based; 7 child and 7 parent units  *Type of use:* self-led | 9–15 year olds with functional abdominal pain | Perceived Impact Satisfaction |
| Nitsch [42] | Healthy Teens @ School to promote healthy lifestyle and reduce eating disorder and obesity risk  *Features:* web-based; 10 weeks  *Type of use:* school-based | 14–19 year olds | Usability |
| Wade [78] | No name  *Features:* web-based; 10 modules  *Type of use:* self-led +/-online therapist support + family involvement | 14–19 year olds with moderate to severe TBI | Acceptability  Perceived Impact Satisfaction |
| **2018** | | | |
| Ahmed [89] | No name  *Features:* mobile-based  *Type of use:* self-led with healthcare professional assistance | 6–11 year olds with apraxia of speech | Acceptability  Satisfaction  Usability |
| Anderson [90] | ITP  *Features:* web-based; 3 modules + daily tracking, messages and videos  *Type of use:* self-led | 7–18 years old with sickle cell disease | Acceptability  Perceived Impact  Satisfaction  Usability |
| Bauermeister [39] | Get Connected to reduce barriers to HIV prevention care  *Features:* web-based  *Type of use:* self-led | 15–24 year old men who have sex with men | Usability |
| Benyakorn [91] | No name  *Features:* web- and tablet-based; 25 sessions  *Type of use:* self-led with parental involvement | 8–17 year olds with ASD | Credibility  Perceived Impact Satisfaction |
| Bernier [40] | NODE  *Features:* mobile-based; 8 modules  *Type of use:* self-led with parental involvement | 4–15 year olds with type 1 diabetes mellitus | Usability |
| Bjureberg [46] | ERITA  *Features:* web-based; 11 modules  *Type of use:* self-led + online healthcare professional involvement | 13–17 year olds with non-suicidal self-injury | Acceptability  Credibility  Perceived Impact Satisfaction |
| Braciszewski [92] | iHeLP  *Features:* web- and mobile-based; single session + daily text messages  *Type of use:* self-led | 18–19 year olds with substance use exiting foster care | Acceptability  Usability |
| Bruggers [9] | Empower Stars!  *Features:* mobile-based; single session  *Type of use:* used in healthcare setting with parent observation | 7–14 year olds with cancer undergoing chemotherapy | Acceptability  Perceived Impact  Satisfaction  Usability |
| Cho [54] | MyPEEPS Mobile for HIV prevention  *Features:* mobile-based; 4 modules  *Type of use:* self-led | 15–18 year old men who have sex with men | Acceptability  Perceived Impact  Satisfaction  Usability |
| De Cock [10] | Snack Track School to improve healthy snacking  *Features:* mobile-based; daily use  *Type of use:* school-based | 14–16 year olds | Acceptability  Satisfaction |
| de la Vega [93] | Fibroline  *Features:* mobile-based; 9 weeks  *Type of use:* self-led | 13–24 year olds with juvenile fibromyalgia syndrome | Acceptability  Usability |
| Grist [94] | BlueIce  *Features:* mobile-based  *Type of use:* self-led | 12–17 year olds who self-harm and attend therapy | Acceptability  Perceived Impact  Satisfaction  Usability |
| Hieftje [95] | PlayForward to reduce sexual risk, substance use, and other risk behaviors  *Features:* tablet-based; as needed  *Type of use:* self-led | 11–14 year olds | Acceptability  Perceived Impact Satisfaction |
| Husted [96] | Young with Diabetes  *Features:* mobile- and tablet-based; daily use  *Type of use:* self-led + healthcare professional encouragement | 15–23 year olds with type 1 diabetes mellitus | Acceptability  Perceived Impact Satisfaction |
| Jolstedt [67, 97] | BiP Anxiety  *Features:* web-based; 12 modules  *Type of use:* self-led + asynchronous support from a healthcare professional | 8–12 year olds with an anxiety disorder | Acceptability  Credibility  Perceived Impact  Satisfaction  User-Reported Adherence |
| Kapitany-Foveny [98] | Once Upon a High for drug use prevention  *Features:* mobile-based; 6 modules  *Type of use:* self-led | High school students | Acceptability  Credibility  Perceived Impact  Satisfaction  Usability |
| Klee [99] | Webdia  *Features:* mobile-based; daily use  *Type of use:* self-led | 10–18 year olds with type 1 diabetes mellitus | Satisfaction  Usability  User-Reported Adherence |
| Leonard [100] | Calm Mom  *Features:* mobile-based + biosensorband; daily use  *Type of use:* self-led | 13–21 year old homeless mothers | Acceptability  Perceived Impact  Satisfaction  User-Reported Adherence |
| Leung [11] | Intervention INC for decreasing childhood obesity risk  *Features:* web- and tablet-based; 6 chapters + weekly text/email messages; parental component  *Type of use:* self-led | 9–12 year old Latinos and black/African Americans | Acceptability  Satisfaction  Usability |
| Lorusso [101] | No name  *Features:* tablet used in healthcare setting; 1 session  *Type of use:* self-led with healthcare professional observation during use | 4–6 year olds with language impairments | Satisfaction  Usability |
| March [12] | BRAVE Self-Help  *Features:* web-based; 10 modules  *Type of use:* self-led | 7–17 year olds with elevated levels of anxiety (2 versions: 7–12 years old & 13–17 years old) | Acceptability  Perceived Impact Satisfaction |
| Parisod  [102, 103]^1^ | Fume for smoking prevention  *Features:* mobile-based  *Type of use:* school-based | 10–13 year olds | Acceptability  Perceived Impact Satisfaction |
| Ramsey [104] | MedaCheck  *Features:* mobile- and tablet-based  *Type of use:* self-led | 13–21 year olds with migraines | Acceptability  Credibility  Perceived Impact  Satisfaction  Usability |
| Richards [63] | eADVICE  *Features:* web-based  *Type of use:* self-led + parental involvement | 6–16 year olds with incontinence | Acceptability  Credibility  Perceived Impact  Usability |
| Sun [55] | Panda for pain management  *Features:* mobile-based  *Type of use:* hospital-based + parental involvement | 12–18 year olds admitted to postsurgical ward | Acceptability  Perceived Impact  Satisfaction  Usability |
| Verberg [105] | The Growth Factory  *Features:* web-based; 6 sessions + phone/email reminders  *Type of use:* self-led + parental involvement | 12–23 year olds with intellectual difficulties | Satisfaction |
| Verdaguer [106] | No name for promoting healthy dietary behaviors to reduce childhood obesity risk  *Features:* web-based; 3 chapters  *Type of use:* self-led + parental involvement | 9–13 year old Latinos and black/African Americans | Acceptability  Satisfaction  Usability |
| Wade [107] | SPAN  *Features:* web- and mobile-based; as needed + 10 weekly coaching sessions  *Type of use:* self-led | 14–22 year olds with acquired brain injury | Acceptability  Satisfaction  Usability |
| **2017** | | | |
| Cai [108] | JIApp  *Features:* mobile-based; daily use  *Type of use:* in healthcare setting | 10–24 year olds with JIA | Acceptability  Perceived Impact  Satisfaction  Usability |
| Castensøe-Seidenfaden [109] | Young With Diabetes  *Features:* web- and mobile-based; daily use  *Type of use:* self-led | 15–22 year olds with type 1 diabetes | Perceived Impact  Usability |
| Dexheimer [14] | SMART  *Features:* web-based; 8 modules  *Type of use:* self-led; separate use by adolescents and parents | 11–18 year olds with mild traumatic brain injury + parents | Acceptability  Usability |
| Goyal [110] | Bant  *Features:* mobile-based; daily use  *Type of use:* self-led | 11–16 year olds with type 1 diabetes | Perceived Impact Satisfaction |
| Hatfield [111] | BOOST-ATM  *Features:* web-based; 4 modules  *Type of use:* self-led | 15–17 year olds with ASD | Acceptability  Perceived Impact |
| Haug [112] | Ready4life  *Features:* mobile-based; texts & booster session  *Type of use:* self-led | 16–19 year olds to prevent substance use | Acceptability  Perceived Impact |
| Holtz [113] | MYT1DHero  *Features:* mobile-based; daily use  *Type of use:* self-led | 10–15 year olds with type 1 diabetes | Acceptability  Satisfaction  Usability |
| Jacobson [36] | No name  *Features:* mobile-based; 2x use during menstruation  *Type of use:* self-led | 13–21 year old girls with heavy menstrual bleeding | Acceptability  Credibility  Usability |
| Jibb [114] | Pain Squad+  *Features:* mobile-based; 2x daily use  *Type of use:* self-led with nurse involvement | 12–18 year olds with cancer pain | Perceived Impact  Satisfaction  Usability |
| Khalil [115] | ASPIRE for smoking prevention  *Features:* web-based; 4 sessions  *Type of use:* self-led | 12–18 year old nonsmokers | Acceptability  Credibility  Perceived Impact Satisfaction |
| Kong [116] | No name  *Features:* mobile-based; prompted use  *Type of use:* self-led | 13–18 year old daily smokers | Acceptability  Satisfaction  Usability |
| Kuosmanen [117] | SPARX-R for preventing depression and improving wellbeing  *Features:* web-based; 7 modules  *Type of use:* school-based | 15–20 year olds in alternative education | Perceived Impact  User-Reported Adherence |
| Lattie [35] | ProjectTECH  *Features:* web-based; 40 lessons  *Type of use:* self-led | 14–19 year olds with depression symptoms who use substances | Acceptability  Perceived Impact  Satisfaction  Usability |
| Lee [118] | Diet-A to promote healthy eating  *Features:* mobile-based; daily use  *Type of use:* self-led | 15–18 year olds | Perceived Impact  Satisfaction  Usability |
| Lolouni [49] | No name  *Features:* web-based; 10 modules  *Type of use:* therapist guided + parent modules | 8–13 year olds with pain-related, functional GI disorders | Acceptability  Perceived Impact Satisfaction |
| Lyles [119] | MYA for healthy body image  *Features:* mobile-based  *Type of use:* school-based | 15–18 year olds | Perceived Impact  Satisfaction  Usability |
| Madeira [44] | Super-Fon  *Features:* tablet-based; 1 session  *Type of use:* therapist guided in healthcare setting | 3–8 year olds with phonological disorders | Satisfaction  Usability |
| McManama O’Brien [120] | Crisis Care  *Features:* mobile-based; adolescent and parent mode; as needed use  *Type of use:* self-led | 13–18 year olds at increased suicide risk after discharge from an acute care setting + parents | Acceptability  Usability  User-Reported Adherence |
| Narad [121] | SPAN  *Features:* mobile-based; as needed use  *Type of use:* self-led + weekly in-person coaching sessions | 14–17 year olds with mild to severe TBI | Acceptability  Satisfaction |
| Navarro [122] | MOVE-IT to promote physical activity  *Features:* web-based; 3x/week  *Type of use:* self-led | 9–15 year olds who are obese | Acceptability  Usability |
| Newton [123] | Check Your Drinking  *Features:* tablet-based; 1 session  *Type of use:* self-led in healthcare setting | 10–17 year olds at the emergency department for an alcohol-related complaint | Acceptability  Perceived Impact Satisfaction |
| Stoll [124] | REACH app  *Features:* mobile-based; 6 sessions  *Type of use:* self-led | 8–12 year olds with anxiety | Acceptability  Credibility  Satisfaction  Usability |
| Thabrew [38] | No name  *Features:* tablet-based; 4-8 modules  *Type of use:* in healthcare setting | 13–18 year olds with long-term physical conditions experiencing anxiety | Acceptability  Usability |
| Turner [13] | b@Ease to promote weight management  *Features:* mobile-based; daily use  *Type of use:* self-led | 14–18 year olds | Acceptability  Usability |
| Widman [125] | ProjectHEARTforGirls.com for HIV/STD prevention  *Features:* web-based; 5 modules  *Type of use:* self-led | Grade 10 girls | Acceptability |
| **2016** | | | |
| Carrasco [74] | Maya  *Features:* web-based  *Type of use:* therapist guided in healthcare setting | 12–18 year old girls with symptoms of depression | Acceptability  Perceived Impact |
| Chapman [126] | Pesky gNATs  *Features:* web-based; 7 sessions  *Type of use:* therapist guided in healthcare setting | 13–18 year olds on a waitlist for CBT for anxiety and/or depression | Perceived Impact  Satisfaction  Usability |
| Danielson [71] | SiHLEWeb.com for HIV/STI prevention  *Features:* web-based; 4 sessions  *Type of use:* self-led | 13–18 year old girls | Acceptability  Usability |
| Glynn [127] | No name  *Features:* tablet-based; 4x/week  *Type of use:* self-led | 7–11 year olds diagnosed childhood absence epilepsy | Perceived Impact  Satisfaction  Usability |
| Hill [57] | LEAP  *Features:* web-based; 2 modules  *Type of use:* self-led | 13–19 year olds who may be experiencing suicidal ideation | Satisfaction |
| Jensen [48] | DailyBurn Tracker for weight control  *Features:* mobile-based; daily use  *Type of use:* self-led | 13–17 year olds with a BMI percentile ≥ 85% | Acceptability  Perceived Impact Satisfaction |
| Kurowski [128] | SMART  *Features:* web-based; 8 modules  *Type of use:* self-led | 11–18 year olds with mild TBI + parents | Acceptability  Perceived Impact Satisfaction |
| Svensson [129] | Dietary assessment app for diet and physical activity  *Features:* mobile-based; daily use  *Type of use:* self-led | 14–16 year olds | Usability |
| Vigerland [65] | DARE Program  *Features:* web-based; 11 modules + parent content  *Type of use:* self-led | 8–12 year olds with specific phobia + parents | Satisfaction |
| Wilansky [47] | No name  *Features:* mobile-based; as needed  *Type of use:* self-led | 12–18 year olds with experience with CBT for anxiety and/or depressive disorders | Satisfaction  Usability |
| **2015** | | | |
| Armbrust [130] | Rheumates@Work  *Features:* web-based; 12 weeks + 4 groups sessions  *Type of use:* self-led and guided in healthcare setting | 8–19 year olds with JIA | Perceived Impact Satisfaction |
| Blackman [131] | 4 smartphone game-based apps to promote physical activity  *Features:* mobile-based; 30 min/day  *Type of use:* self-led | Middle & high school students from low-SES or of racial/ethnic minority | Perceived Impact Satisfaction |
| Brady [132] | TeensTalkHealth  *Features:* web-based; 8 modules Sexual health promotion  *Type of use:* self-led | 14–18 year olds | Acceptability  Credibility  Satisfaction  Usability |
| Bul [133] | Plan-It Commander  *Features:* web-based; up to 8x/week  *Type of use:* self-led | 8–12 year olds with ADHD | Acceptability  Perceived Impact  Usability |
| Cox [134] | Cogmex  *Features:* web-based; 25 sessions  *Type of use:* self-led | 8–16 year old survivors of pediatric brain tumors | Acceptability  Perceived Impact  Satisfaction  Usability |
| Doumas [77] | eCHECKUP TO GO to prevent problem drinking  *Features:* web-based; 1 session  *Type of use:* school-based | 9^th^ grade students | Perceived Impact  Satisfaction  Usability |
| Enah [135] | Fast Car: Travelling Safely around the World for HIV prevention  *Features:* web-based; 1 session  *Type of use:* self-led | 12–16 year olds living in rural areas | Acceptability  Satisfaction |
| Gladstone [76] | CATCH-IT  *Features:* web-based; 14 modules & 4 parent modules  *Type of use:* therapist support in  healthcare setting | 14–21 year olds with elevated levels of depressive symptoms | Perceived Impact  Usability |
| Kenny [136] | CopeSmart for mental health promotion  *Features:* mobile-based; regular use  *Type of use:* self-led | 15–17 year olds | Perceived Impact  Satisfaction  Usability |
| Kobak [34, 137] | No name  *Features:* tablet- and mobile-based; 12 weeks with 2 texts/day  *Type of use:* healthcare setting | 12–17 year olds with a DSM-5 mood disorder | Usability |
| Law [60] | Web-MAP  *Features:* web-based; 8 modules; family-based  *Type of use:* self-led with therapist coaching | 11–17 year olds with recurrent headache | Acceptability  Perceived Impact Satisfaction |
| Marsac [138] | Coping Coach  *Features:* web-based; 3 levels  *Type of use:* healthcare setting with parental support | 8–12 year olds with a traumatic medical event within the past 60 days + parents | Acceptability  Credibility  Satisfaction  Usability |
| Marsch [139] | The Therapeutic Education System  *Features:* web-based; 26 modules  *Type of use:* healthcare setting | 12–18 year olds with a SUD | Acceptability  Perceived Impact  Usability |
| Moulos [37] | SPLENDID  *Features:* mobile-based + wearable sensors  *Type of use:* self-led | 15–17 year olds who are obese or have an eating disorder | Usability |
| Mustanski [140] | Queer Sex Ed for sexual health promotion  *Features:* web-based; 5 modules  *Type of use:* self-led | 16–20 year olds | Acceptability |
| Palermo [72] | Web-MAP  *Features:* web-based; 8 modules; family-based  *Type of use:* self-led in conjunction with therapy | 12–17 year olds with chronic pain | Acceptability  Satisfaction |
| Pretlow [141] | No name  *Features:* mobile-based; daily use  *Type of use:* self-led | 10–21 year olds who are obese | Perceived Impact |
| Sousa [142, 143] | Next.Step  *Features:* web-based; 10 modules  *Type of use:* self-led | 12–18 year olds with a BMI ≥95^th^ percentile | Usability  User-Reported Adherence |
| Sze [144] | MAMRT  *Features:* web-based; daily use  *Type of use:* self-led and in healthcare setting | 8–12 year olds who are overweight + parents | Perceived Impact  Usability |
| Voerman [145] | Move It Now  *Features:* web-based; 7 modules  *Type of use:* therapist guided | 12–17 year olds with chronic pain | Satisfaction |
| Williamson^2^ [146, 147] | YP Face IT  *Features:* web-based  *Type of use:* self-led with parent support | 12–19 year olds with appearance-related anxiety as a result of a visible difference and their parents | Acceptability |
| Wozney [148] | Breathe  *Features:* web-based; 8 modules  *Type of use:* self-led | 15–24 year olds involved in peer advocacy for mental health and illness | Usability |
| **2014** | | | |
| Bannink [149] | E-health4U to improve general health, growth and development  *Features:* web-based; 1 session + email follow-up  *Type of use:* school-based | 12–18 year olds | Acceptability  Credibility  Perceived Impact  Satisfaction  Usability |
| Breakey [150] | Teens Taking Charge: Managing Hemophilia Online  *Features:* web-based; 8 modules  *Type of use:* self-led | 12–18 year olds with hemophilia transitioning from pediatric to adult care | Satisfaction |
| Comer [151] | No name  *Features:* web-based; 12 sessions  *Type of use:* family-based with therapist involvement | 4–8 year olds with OCD + parents | Satisfaction |
| Gladstone [75] | CATCH-IT  *Features:* web-based; 14 modules & 4 parent modules  *Type of use:* therapist support in  healthcare setting | 14–21 year olds with elevated levels of depressive symptoms | Perceived Impact  Usability |
| Hetrick [152] | Reframe-IT  *Features:* web-based; 8 modules  *Type of use:* school-based | 14–18 year olds with past month suicidal ideation | Perceived Impact |
| Lubans [153] | ATLAS  *Features:* web- and mobile-based; as needed  *Type of use:* school-based | 7^th^ grade boys in low-income areas at risk for obesity | Satisfaction  Perceived Impact  Usability |
| Manicavasagar [154] | Bite Back for general mental health promotion  *Features:* web-based; 1 hour/week  *Type of use:* self-led | 12–18 year olds | Acceptability  Satisfaction  Usability |
| Newton [155] | Climate Schools: Alcohol and Cannabis Course  *Features:* web-based; 6 lessons  *Type of use:* school-based | 13–14 year olds | Acceptability  Perceived Impact  Satisfaction |
| O’Malley [69] | Reactivate  *Features:* mobile-based  *Type of use:* self-led | 12–17 year olds who are obese | Acceptability  Satisfaction  Usability |
| Ybarra [156] | CyberSenga for HIV prevention  *Features:* web- based; 5 session + 1 booster session  *Type of use:* self-led | 13–19 year olds | Acceptability  Usability |
| **2013** | | | |
| Ezendam [157] | FATaintPHAT for preventing excessive weight gain  *Features:* web-based; 8 sessions  *Type of use:* school-based | 12–13 year olds | Satisfaction  Perceived Impact  Usability  User-Reported Adherence |
| Haug [158] | Ready4life to prevent problem drinking  *Features:* web- and mobile-based; 12 weeks  *Type of use:* self-led | 16–20 year olds | Acceptability  Perceived Impact Satisfaction |
| Hardy [159] | CogmedRM  *Features:* web-based; 25 sessions  *Type of use:* self-led | 8–16 year old survivors of pediatric brain tumors, ALL | Acceptability  Satisfaction  User-Reported Adherence |
| Harrell [160] | BrainWorks™  *Features:* web-based; 4x/week  *Type of use:* self-led | 12–17 year olds with chromosome 22q11.2 deletion syndrome | Acceptability  Perceived Impact  Satisfaction  Usability  User-Reported Adherence |
| Ritterband [161] | U-CAN-POOP-TOO  *Features:* web-based; 3 modules + follow up  *Type of use:* self-led + parental support in conjunction with standard medical care | 5–12 year olds with pediatric encopresis + parents | Usability |
| Spook [162] | mEMA app for improving diet and physical activity  *Features:* mobile-based; daily use  *Type of use:* self-led | 16–21 year olds | Acceptability  Satisfaction  Usability |
| Vigerland [66] | DARE Program  *Features:* web-based; 11 modules + parent content  *Type of use:* self-led with parental support | 8–12 year olds with specific phobia + parents | Satisfaction |
| **2012** | | | |
| Bradley [163] | Feeling Better Program  *Features:* web-based; 12 modules  *Type of use:* self-led | 15–18 year olds with no previous mental illness diagnosis | Acceptability  Perceived Impact |
| Lau [164] | Teen-step.com for promotion of physical activity  *Features:* web- and mobile-based; 4 modules  *Type of use:* self-led | 12–15 year olds | Acceptability  Credibility  Satisfaction  Usability |
| Shegog [165] | +CLICK  *Features:* web-based  *Type of use:* healthcare setting | 13–24 year olds who are HIV positive | Acceptability  Credibility  Usability |
| Whittaker [166] | MEMO *Features:* web- and mobile-based; 9 weeks  *Type of use:* self-led | 13–17 year olds without depression or risk of self-harm | Satisfaction  Perceived Impact |
| **2011** | | | |
| Coyle [167] | gNAts Island  *Features:* web-based; 6 sessions  *Type of use:* therapist guided in  healthcare setting | 11–16 year olds experiencing anxiety difficulties | Acceptability  Perceived Impact Satisfaction |
| Iloabachie [168] | CATCH-IT  *Features:* web-based; 14 modules & 4 parent modules  *Type of use:* therapist support in  healthcare setting | 14–21 year olds with persistent subclinical depression | Acceptability  Perceived Impact  Usability |
| Marsch [169] | No name (for prevention of HIV, STIs and hepatitis)  *Features:* web-based; 25 modules  *Type of use:* in healthcare or community setting | 12–18 year olds with a SUD | Satisfaction |
| Tillfors [170] | No Name  *Features:* web-based; 9 modules  *Type of use:* self-led | 15–21 year olds with social anxiety disorder | Satisfaction |
| **2010** | | | |
| Newton [171] | Climate Schools: Alcohol and Cannabis Course  *Features:* web-based; 6 lessons  *Type of use:* school-based | 8^th^ grade students | Perceived Impact Satisfaction |
| Stinson [172] | Teens Taking Charge: Managing Arthritis On-line  *Features:* web-based; 12 modules  *Type of use:* self-led | 12–18 year olds with JIA | Satisfaction  Usability |
| **2009** | | | |
| Markham [173] | +CLICK  *Features:* web-based; 1x use  *Type of use:* in healthcare setting | 13–24 year olds who are HIV positive | Credibility  Satisfaction  Usability |
| Landback [174] | CATCH-IT  *Features:* web-based; 14 modules & 4 parent modules  *Type of use:* therapist support in  healthcare setting | 14–21 year olds with sub-threshold depression | Perceived Impact  Satisfaction  Usability |
| Long [175]  Palermo [73] | Web-MAP  *Features:* web-based; 8 modules; family-based  *Type of use:* self-led | 11–17 year olds with chronic idiopathic pain | Acceptability  Satisfaction  Usability |
| **2008** | | | |
| O’Conner-Von [176] | Tonsils! Who Needs ’em?  *Features:* web-based  *Type of use:* self-led | 10–16 year olds with anxiety regarding tonsillectomy procedures | Satisfaction  Usability |
| Ritterband [177] | U-CAN-POOP-TOO  *Features:* web-based; 3 modules + follow up  Type of use: self-led + parental support in conjunction with standard medical care | 5–12 year olds diagnosed with pediatric encopresis | Acceptability  Perceived Impact  Satisfaction  Usability |

ADHD: attention-deficit hyperactivity disorder; ALL: acute lymphoblastic leukemia; ASD: Autism Spectrum Disorder; BMI: body mass index; CBT: cognitive-behavioral therapy; DSM: Diagnostic and Statistical Manual of Mental Disorders 5^th^ edition; GI: gastrointestinal; HIV: human immunodeficiency virus; JIA: juvenile idiopathic arthritis; OCD: Obsessive-Compulsive Disorder; SUD: substance use disorder; TBI: traumatic brain injury; HIV: human immunodeficiency virus; SES: socio-economic status; STI: sexually transmitted infection; SUD: substance use disorder; PrEP: pre-exposure prophylaxis

^1^Pilot evaluation published year prior

^2^Study protocol published year prior
