# Supplementary material for: Establishing a Working Definition of User Experience for eHealth Interventions of Self-reported User Experience Measures With eHealth Researchers and Adolescents: Scoping Review
Source: J Med Internet Res. 2021 Dec 2;23(12):e25012. doi: 10.2196/25012 (PMC8686463; doi:10.2196/25012)
Supplement: Multimedia Appendix 6 [file jmir_v23i12e25012_app6.docx]

## Multimedia Appendix 6

Results from the quality assessment of user experience measures used in the 129 studies included in the review. Green: met criterion; Yellow: partially met criterion.

| **Measures assessed to be ‘well-established’** | | | | |
| --- | --- | --- | --- | --- |
|  | The measure must have been presented in at least two peer-reviewed articles by different investigators or investigatory teams | Sufficient detail about the measure to allow critical evaluation and replication | Detailed information indicating good to very strong/excellent validity and reliability in at least one peer-reviewed article | eHealth study use |
| SUS |  |  |  | [14, 34-42] |
| SUS (Portuguese version) |  |  |  | [44] |
| CEQ |  |  |  | [46] |
| CSQ-8 |  |  |  | [46-49] |
| GEQ |  |  |  | [7, 10] |
| PSSUQ (CSUQ)^1^ |  |  |  | [54, 55] |
| SSS |  |  |  | [57] |
| TEI-SF |  |  |  | [60] |
| USE |  |  |  | [35] |
| WAI-SR |  |  |  | [46, 63] |
| **Measures assessed to be ‘approaching well-established’** | | | | |
|  | The measure must have been presented in at least two peer-reviewed articles, which might be by the same investigator or investigatory team | Sufficient detail about the measure to allow critical evaluation and replication | Validity *or* reliability information was missing or presented in vague terms, or included poor to moderate values | eHealth study use |
| CSS |  |  |  | [65-67] |
| SUMI |  |  |  | [69] |
| WAMMI |  |  |  | [71] |
| Author-adapted TEI-SF |  |  |  | [72, 73] |
| Author-developed questionnaires |  |  |  | [75, 76] |
| **Measures assessed to be ‘promising’** | | | | |
|  | The measure was presented in only one peer-reviewed article | Sufficient detail about the measure to allow critical evaluation and replication | Validity *or* reliability information missing or presented in vague terms, or included poor to moderate values | eHealth study use |
| Adapted and combined USE & RPS |  |  |  | [124] |
| Author-developed Process Evaluation Questionnaire |  |  |  | [157] |
| Study specific questionnaire adapted from another author-developed, study specific questionnaire |  |  |  | [115] |
| Author-developed questionnaires |  |  |  | [100] |
|  |  |  |  | [74] |
|  |  |  |  | [77] |
|  |  |  |  | [78] |
|  |  |  |  | [134] |
|  |  |  |  | [132] |
|  |  |  |  | [177] |
|  |  |  |  | [161] |
|  |  |  |  | [164] |
|  |  |  |  | [122] |
| **Measures assessed to be ‘not yet established’** | | | | |
|  | The measure must have been presented in at least one peer-reviewed article | There is sufficient detail about the measure to allow critical evaluation and replication | No validity and reliability information presented | eHealth study use |
| Adapted AES |  |  |  | [114] |
| Adapted English (& German) CSQ-8 (ZUF-8) |  |  |  | [151] [87] |
| Adapted USE |  |  |  | [108] |
| Adapted SUS |  |  |  | [120] |
| Adapted SUS |  |  |  | [119] |
| Combined & author-adapted SUS, USE, and another author-developed, study specific tool |  |  |  | [11, 106] |
| Combined & author-adapted SUS & SUPR-Q |  |  |  | [148] |
| Adapted and translated SRS (Dutch) |  |  |  | [105] |
| Author-developed questionnaires |  |  |  | [8, 9, 13, 36, 37, 41, 44, 88-91, 94, 95, 97-99, 101-105, 107, 109-112, 119, 121, 123, 126, 127, 130, 133, 136, 138-140, 144, 145, 149, 150, 152, 154, 156, 158-160, 166, 167, 169, 170, 175] |
|  |  |  |  | [14, 47, 92, 113, 116, 128, 141-143, 155, 171] |
| Study specific questionnaire adapted from another author-developed, study specific questionnaire |  |  |  | [12, 117, 118, 125, 162, 165, 168, 173, 174] |
|  |  |  |  | [153] |
| Author-developed interviews |  |  |  | [96, 108, 129, 131, 135, 146, 163, 172, 176] |
|  |  |  |  | [8, 35, 42, 47, 88, 93, 95, 138, 148] |

^1^The CSUQ and PSSUQ are the same questionnaire; the only difference is the CSUQ wording is appropriate for use in field settings or surveys rather than in a scenario-based usability evaluation [53, 64]

SUS: System Usability Scale; CSQ-8: Client Satisfaction Questionnaire-8; SSS: Satisfaction with Services Scale; TEI-SF: Treatment Evaluation Inventory - short form; USE: Usefulness, Satisfaction and Ease of use questionnaire; CSS: Client Satisfaction Scale; SUMI: Standardized Software Usability Measurement Inventory; WAMMI: Website Analysis and Measurement Inventory; RPS: Reactions to Program Scale; AES: Acceptability E-Scale; SUPR-Q: Standardized User Experience Percentile Rank Questionnaire; CEQ: Credibility/Expectancy Questionnaire; WAI-SR: Working Alliance Inventory – Revised Short Version; PSSUQ: Post-Study System Usability Questionnaire; GEQ: Game Experience Questionnaire: SRS: Sessions Rating Scale; CSUQ: Computer Systems Usability Questionnaire
