# Supplementary material for: Establishing a Working Definition of User Experience for eHealth Interventions of Self-reported User Experience Measures With eHealth Researchers and Adolescents: Scoping Review
Source: J Med Internet Res. 2021 Dec 2;23(12):e25012. doi: 10.2196/25012 (PMC8686463; doi:10.2196/25012)
Supplement: Multimedia Appendix 7 [file jmir_v23i12e25012_app7.docx]

## Multimedia Appendix 7

Evaluation measures used in eHealth studies assessed to be ‘promising’.

| **Measure and targeted user experience** | **Features** | **Psychometric Properties** | | **eHealth study** |
| --- | --- | --- | --- | --- |
|  |  | **Validity** | **Reliability** |  |
| Adapted and combined USE & Reactions to Program Scale (RPS)  Acceptability Satisfaction Credibility Usability | 26 items  10-point Likert scale  *Administration:*  NR | NR | *Internal Consistency* eHealth study sample [124]:  Ease of use, α=.90 Quality of support information, α=.78  System ease of learning, α=.91  System satisfaction, α =.88  Stigma, α=.83  Overall usability, α=.93 | [124] |
| Study specific tool adapted from another author-developed, study specific tool  Acceptability  Perceived Impact  Satisfaction | 25+ items  10-point Likert scale  *Administration:* web-based | NR | *Internal Consistency*  eHealth study sample [115]:  Perceived interactivity α=.94  Perceived entertainment α=.92  Perceived presence α=.88  Emotional involvement α=.55 | [115] |
| Author-developed Process Evaluation Questionnaire  Satisfaction Impact User-Reported Adherence Usability | 24 items  Likert type scales  *Administration:*  NR | NR | *Internal Consistency*  eHealth study sample [157]:  Linking and personal relevance, α=.91  Usefulness of information, α=.92 | [157] |
| Author-developed questionnaire  Acceptability Perceived Impact  Satisfaction Usability | 11 (adolescent) 20 (parent) items  5-point Likert scale  *Administration:* NR | NR | *Inter-Rater Reliability*  eHealth study sample [134]:  κ=.07–.40 (mean .23) | [134] |
| Author-developed questionnaire  Acceptability Credibility Satisfaction Usability | 26 items  5-point Likert scale  *Administration:* web-based | NR | *Internal Consistency*  eHealth study sample across different time-points [132]:  Perceived engagement,  α=.55–.74  Comfort on website,  α=.61–.67  Accessibility/ease of use, α=.59–.70  Personal relevance of content, α=.71–.82  Credibility of health educators, α=.77–.85 | [132] |
| Author-developed questionnaire  Acceptability Perceived Impact Satisfaction Usability | 70 items  3- and 5-point Likert scales + open-ended  *Administration:* telephone-based | NR | *Internal Consistency*  eHealth study sample [177]:  Physical symptoms, α=.88  Comfort, α=.80  Worry/mood, α=.65  School/social support, α=.94  Cost/time, α=.64 Overall α=.69 | [177] |
| Author-developed questionnaire  Usability | 12 items  5-point Likert scale + open-ended  *Administration:* NR | NR | *Internal Consistency*  Questionnaire development sample [177]:  α=.69 | [161] |
| Author-developed questionnaire  Acceptability Satisfaction Credibility Usability | 11 items  5-point Likert scale + close-ended  *Administration:*  paper-based | NR | *Internal Consistency*  eHealth study sample [164]:  Web contents (5 items), α=.92  SMS contents (4 items), α=.91 | [164] |
| Author-developed questionnaire  Acceptability Usability | 20 items  5-point Likert scale  *Administration:*  paper-based | NR | *Internal Consistency*  eHealth study sample [122]:  Acceptance, α=.81  Usability, α=.42 | [122] |
| Author-developed questionnaire  Acceptability Satisfaction Perceived Impact User-reported adherence | 8 items  4-point Likert scale  *Administration:* NR | NR | *Internal Consistency*  eHealth study sample [100]: α=.89 | [100] |

NR: not reported
